# Correlates of health-related quality of life in primary caregivers of perinatally HIV infected and HIV exposed uninfected adolescents at the Kenyan Coast

**DOI:** 10.1186/s12955-022-01915-z

**Published:** 2022-01-21

**Authors:** Patrick N. Mwangala, Derrick Ssewanyana, Paul Mwangi, Esther Chongwo, Carophine Nasambu, Vincent A. Kagonya, Gaia Scerif, Charles R. Newton, Amina Abubakar

**Affiliations:** 1 Centre for Geographic Medicine Research Coast, Kenya Medical Research Institute (KEMRI), P.O. Box 230-80108, Kilifi, Kenya; 2 School of Public Health, University of the Witwatersrand, 27 St Andrews Road, Parktown, 2193 South Africa; 3 Department of Experimental Psychology, University of Oxford, Anna Watts Building, Oxford, OC2 6GG UK; 4 Department of Psychiatry, Warneford Hospital, University of Oxford, Warneford Ln, Oxford, OX3 7JX UK; 5 Department of Public Health, Pwani University, P.O. Box 195-80108, Kilifi, Kenya; 6 Institute for Human Development, Aga Khan University, P.O. Box 30270-00100, Nairobi, Kenya

**Keywords:** HIV, Primary caregivers, Adolescents, Health-related quality of life, Kenya

## Abstract

**Background:**

Mothers and other primary caregivers play a crucial role in looking after perinatally HIV infected, and HIV exposed uninfected adolescents in sub-Saharan Africa. Day- to-day caregiving in the context of limited instrumental support and added biomedical risk (HIV seropositivity) may expose these caregivers to adverse states of health. Unfortunately, very few studies have examined their health-related quality of life (HRQoL). Our study documents the HRQoL profile, and associated factors in primary caregivers of perinatally HIV infected, perinatally HIV exposed but uninfected and HIV unexposed/uninfected adolescents aged 12–17 years at the Kenyan Coast.

**Methods:**

This was a cross-sectional analysis of 485 primary caregivers: 195 of perinatally HIV infected adolescents, 128 of perinatally HIV exposed but uninfected adolescents and 162 of HIV unexposed/uninfected adolescents. All caregivers completed a self-report measure of HRQoL (having 8 subscales), depressive symptoms, and parenting stress. They also provided their sociodemographic information and that of the care recipients. We used one-way analysis of variance to assess statistical differences among the groups. Linear regression analyses were used to identify correlates of HRQoL.

**Results:**

Overall, caregivers of HIV unexposed/uninfected adolescents reported significantly higher mean HRQoL scores than the other caregivers in the overall HRQoL domain and majority of the subscales. There were no statistical differences in the overall HRQoL scores and most subscales between caregivers of HIV exposed adolescents**.** Linear regression analyses across the sample indicated that depressive symptoms, increasing age of caregiver, and caring for an adolescent perinatally exposed to HIV were significantly associated with reduced HRQoL at both the overall and sub-scale level. Having a professional job relative to subsistence farming was the only factor associated with improved overall HRQoL. At subscale level, higher socioeconomic status correlated positively with HRQoL while being a grandparent, level of education, parenting stress were negatively associated with HRQoL.

**Conclusions:**

Caregivers in this sample, especially those who are ageing, at risk of mental ill-health, and taking care of adolescents perinatally exposed to HIV, appear to be vulnerable to poor quality of life. Inclusive and multi-component interventions tailored to the caregivers' psychosocial and mental needs will potentially enhance their quality of life.

**Supplementary Information:**

The online version contains supplementary material available at 10.1186/s12955-022-01915-z.

## Background

By the end of 2020, approximately 1.8 million adolescents (10–19 years of age) were living with HIV worldwide, including 0.7 million younger adolescents aged 10–14 years (49% female) and 1.1 million older adolescents aged 15–19 years (60% female) [1]. The majority (88%) of these adolescents are from sub-Saharan Africa (SSA) [1]; thus, the health-related quality of life (HRQoL) of these adolescents, as well as that of their caregivers, is of public health importance. Overall, this adolescent age group represents a mixture of young people perinatally infected with HIV and those more recently infected (horizontal infection). About 65% of older female adolescents living with HIV acquire it horizontally compared to only 43% among older male adolescents living with HIV. These sex differences are especially prominent in SSA, where 62% of older female adolescents living with HIV acquired it horizontally, compared with 28% of older male adolescents living with HIV. Unlike in the early 2000s, when perinatally infected children had high mortality during infancy, and poor survival beyond childhood, the era of potent antiretroviral therapy (ART) has registered a dramatic survival of children living with HIV, with many (as high as 90%) getting into adolescence and adulthood [2, 3]. Amidst this unprecedented breakthrough in HIV care, the SSA region still experiences challenges in providing long-term care and support for the many adolescents living with HIV [4] as well as those perinatally exposed to maternal HIV but uninfected who have been shown to have substantially higher morbidity and mortality compared with children born to uninfected mothers [5].

In the current era of widespread effective HIV treatment and improved life expectancy, most of the primary caregivers of adolescents perinatally exposed to HIV in SSA are young biological parents, frequently mothers who are living with HIV themselves [6, 7]. Typically, such caregivers assume the total care of these adolescents with limited formal and informal support systems [8, 9]. The majority of the existing literature on caregiving of children and adolescents perinatally exposed to HIV in SSA depicts it as burdensome with serious economic, psychological, and social strain [8–10] and often associated with suboptimal child outcomes [11]. However, other studies suggest that caregiving in the context of HIV is associated with intrinsic and extrinsic rewards, including emotional and psychological well-being, better health status, higher satisfaction and higher quality of life among the carers [12, 13].

Health-related quality of life (HRQoL) has emerged as a salient indicator of HIV care and an essential target for HIV-related research, aimed at expanding the continuum of service [14]. HRQoL is a multidimensional measure of subjective overall health and well-being, including mental, social, and physical aspects of functioning [15]. Among individuals living with HIV, research has shown that HRQoL can be affected by several sociodemographic, psychosocial, and biomedical factors [15]. Both cross-sectional and longitudinal studies have consistently shown that social support, increased physical activity, higher socioeconomic status, better nutrition and virologic recovery are associated with improved HRQoL in people living with HIV [15]. On the other hand, comorbidities (such as depression), stigma, drug and substance use have been associated with declining HRQoL [15]. Unfortunately, the evidence base on HRQoL among adolescents perinatally exposed to HIV, and their caregivers in SSA is insufficient and poorly documented. The few studies attempting to examine the HRQoL of caregivers of children and adolescents perinatally exposed to HIV have focussed on isolated components of HRQoL (mostly socioemotional well-being) [11, 16–20] without documenting the correlates of HRQoL. Many of these studies tend to aggregate the HRQoL outcomes for carers of children and adolescents perinatally exposed to HIV respectively, despite the apparent differences in their care needs [11, 12, 16–21].

Globally, it is recognized that perinatally HIV infected adolescents experience poorer HIV-related outcomes than young children, including high mortality, poorer retention in care and virologic treatment failure [22]. Besides, the adolescents have to deal with long-term complications of therapy, sexual and reproductive health, mental health challenges, and higher education and career training [22]. For children perinatally exposed to HIV but uninfected, research indicates that they are potentially at risk of poor health outcomes, including impaired growth and neurodevelopment and a higher risk of morbidity and mortality from infectious disease [23]. The adolescents perinatally exposed to HIV but uninfected may also face certain challenges such as orphanhood, living with and caring for sick household members [24]. At times, these adolescents may also experience elevated vulnerability for engagement in risky behaviours [25]. For caregivers, many have to bear the costs of care and treatment for family with little or no support, and sometimes provide care while ill and coping with the unique demands of adolescent caregiving. Optimizing the care for these caregivers, therefore, requires an understanding of the factors that contribute to their HRQoL. This knowledge will help healthcare providers and policymakers to devise evidence-based interventions and guidelines for improving the quality of care and wellbeing for both the adolescents perinatally exposed to HIV and their caregivers.

In Kenya, the situation is not different. To our knowledge, no study has examined the HRQoL of caregivers of adolescents perinatally exposed to HIV in the country. The few studies investigating caregiving in the context of HIV have addressed issues such as the evaluation of support groups for guardians of orphans and vulnerable children [26], HIV-related stigma [27], HIV status disclosure [28], the prevalence of anxiety and depression among caregivers of children living with HIV [29]. Therefore, the current study seeks to expand the evidence base on the HRQoL among primary caregivers of adolescents perinatally exposed to HIV residing in the rural settings of Kenya. Specifically, we aim to examine the status of HRQoL among 195 caregivers of perinatally HIV infected adolescents (PHI), 128 caregivers of perinatally HIV exposed but uninfected adolescents (PHEU) and 162 caregivers of HIV unexposed/uninfected adolescents (HUU). We hypothesized that caregivers of adolescents living with HIV would have lower mean HRQoL scores than caregivers of adolescents perinatally exposed to HIV but uninfected and caregivers of adolescents not exposed to HIV infection.

## Methods

### Study design and setting

This work is part of baseline data collected from an ongoing larger observational cohort study, the Adolescent Health Outcomes Study, examining different health outcomes, e.g., cognition, mental health, and their psychosocial environment in adolescents perinatally exposed to HIV. The baseline data was conducted between November 2017 and October 2018. The current paper is an analytical study of the quality of life of the caregivers of the adolescents who participated in the baseline phase of the main study. The study was conducted at the Centre for Geographic Medicine Research-Coast at the Kenya Medical Research Institute (CGMRC-KEMRI) located in Kilifi County. With about 1.4 million people, most of the residents in Kilifi county are rural dwellers (61%) [30]. Moreover, nearly half of this population is composed of children below the age of 15 years [31]. By the end of 2017, Kilifi county had an overall HIV prevalence of 4%, and approximately 2,511 adolescents (10–19 years) living with HIV [32].

### Sample size

The sample size target for the primary study was 600 adolescents (200 in each arm) together with their caregivers. The sample size computation was based on the effects sizes of a previous study conducted in Kilifi on neuropsychological profiles of HIV affected adolescents. This sample size accounted for a potential 20% attrition in the 3-year follow up. Considerations were also made to yield stable models of structural equation modelling, which required at least 200 participants in the adolescent outcomes. The sample size of 600 adolescents gave more than 95% power to detect differences in the main outcomes between groups. The final sample size was (558 adolescents—200 HUU, 157 HEU and 201 living with HIV and together with their caregivers). Further details on this have been described elsewhere [33].

### Participants and recruitment procedures

Primary caregivers of PHI and PHEU adolescents (12–17 years) were recruited using sequential sampling of all families attending specialized HIV clinics from eight HIV treatment and care clinics within Kilifi County. Recruitment was carried out by a trained research assistant in collaboration with experienced healthcare workers attached to the participating HIV treatment facilities. Additionally, some of the primary caregivers of PHEU adolescents were recruited by conducting home visits to families affected with HIV within their community with the help of a community health worker stationed at an HIV clinic. On the other hand, primary caregivers of HUU adolescents were randomly sampled among different households within the Kilifi Health and Demographic Surveillance System (KHDSS) using the KHDSS population register [34].

The caregivers had to be the adolescents' primary carers and willing to accompany them for assessments. Carers of PHI adolescents needed to have fully disclosed their HIV status to the adolescents. They also needed to be aware of their own HIV status. The HIV status of PHEU adolescents was confirmed by checking the maternal medical records (antenatal care cards) which confirmed HIV infection of the mother during pregnancy. Recent medical records of the adolescent (if available) were also used to verify the status. The selection was limited to caregivers who willingly shared their HIV test results at the time of their pregnancy with the adolescents receiving care. HIV unexposed and uninfected adolescents were not directly tested for HIV. However, recruitment was restricted to those whose mothers willingly shared their HIV test results at the time of their pregnancy with the adolescent. For both caregivers of PHEU and HUU adolescents, a brief screening checklist was utilized to exclude caregivers whose adolescents had experienced severe childhood illness or were having recurring health problems to minimize the possibility of including caregivers of HIV-infected adolescents in these two groups. The HIV status of primary caregivers was not ascertained by HIV testing in the current study. Among caregivers of perinatally exposed adolescents, their status was confirmed by checking the maternal medical records (antenatal care cards) which confirmed HIV infection of the mother during pregnancy. For the remaining caregivers (including those of HUU adolescents), we used HIV screening questions (e.g., whether they are HIV infected and on antiretroviral treatment) to ascertain their HIV status. More details of the study procedures are described elsewhere [33].

### Measures

Several measures were utilised in the main study. In this present study, we focused on only the specific measures that are relevant for the assessment of HRQoL and plausible correlated factors as described below.

#### Sociodemographic and socioeconomic information

Data on caregivers' age, sex, education level, marital status, occupation, religion, HIV status and relationship to the adolescent receiving care was recorded. Caregivers' socioeconomic status was measured using an asset index that has previously been used in Kilifi [35]. The asset index items screened for the ownership of a list of disposable assets by participants (or their family) such as radio, television, bicycle, and motorbike. A single index score of socioeconomic status was generated, with a higher score indicating a higher socioeconomic status. Additionally, information on adolescents' age, sex, current educational level, grade retention, and orphanhood status was captured and ascertained in the presence of their caregiver and from other records, including birth certificates. We also measured adolescents' mid-upper arm circumference, head circumference, height and weight following recommended procedures [36, 37]. Height (in metres) and weight (kgs) were used to generate body mass index (BMI).

#### Caregivers' mental health assessment

*Depressive symptoms:* The Patient Health Questionnaire (PHQ-9*)* was used to assess caregivers' depressive symptoms [38]. This is a self-report depression severity measure, which has been applied in multiple cultural settings yielding good psychometric properties. The measure is scored on a 4-point Likert scale from '0' (not at all) to '3' (nearly every day) with total scores ranging from 0 to 27. PHQ-9 has previously been used in the study setting in Kenya, yielding good psychometric properties [39]. This tool was translated and back-translated into Swahili using WHO guidelines then pre-tested in the study setting before being used in the current study. In this study, its internal consistency was 0.86 (*95% confidence interval 0.83–0.89*). For analysis purposes, we used the continuous scores of the tool. Increasing scores on the measure translate into increasing depressive symptoms.

#### Parenting stress

*The parental stress scale (PSS)* was used to measure the levels of stress experienced by caregivers [40]. It has 18 self-report items which take into account both positive and negative aspects of caregiving. Respondents agree or disagree on various items about their typical relationship with their child on a 5-point Likert scale from '0' (strongly disagree) to '4' (strongly agree) with total scores ranging from 0 to 72. The higher the score, the higher the measured level of caregiving stress. This tool was translated and back-translated into Swahili using World Health Organization (WHO) guidelines then pre-tested in the study setting before being used in the current study. In the present analysis, its internal consistency was 0.78 (*95% confidence interval 0.75–0.81*). For analysis purposes, we used the continuous scores of the tool.

#### Health-related quality of life

We assessed HRQoL using the RAND 36-Item Health Survey 1.0 [41]. This is a generic measure of self-reported HRQoL assessing 8 health domains, namely: (i) *physical functioning*; (ii) *bodily pain*; (iii) *role limitations due to physical health problems*; (iv) *role limitations due to emotional problems*; (v) *emotional well-being*; (vi) *social functioning*; (vii) *energy/fatigue* (4 items); and (viii) *general health*. This measure has been previously used in the current study setting [42, 43]. Final scores in the different subscales range from 0 to 100. In the current study, this measure yielded an internal consistency value of 0.88 (*95% confidence interval 0.86–0.89*). More details of the description of this tool are provided in the supplementary files (Additional file 3).

### Data analysis

Initial data checks were conducted, and all missing values noted. Box plots and histograms were plotted to assess the normality of continuous variables. For categorical variables, proportions and percentages were generated to explore their distribution. One-way analysis of variance (ANOVA) was used to identify group differences on normally distributed continuous variables. At the same time, the Kruskal–Wallis H test was utilised to identify group differences on skewed continuous variables. Pearson's chi-squared test was utilised to identify group differences in categorical variables. Fisher's exact test was used for categorical values which have some of their cell values less than 5. Univariable linear regression analyses were conducted to identify correlates/factors associated with the HRQoL variables among caregivers. All factors from the univariable analysis having a P value ≤ 0.20 [44] were then entered into a multivariable linear regression model to examine the independent correlates through a backward elimination process, in which the largest P-value was excluded till all remaining variables make significant contributions to the final model by considering the t test and F test [45]. We tested for model assumptions for all the linear regression models used in the analysis (linearity, homoscedasticity, normality). Multicollinearity was checked using variance inflation factor. After these analyses, we also conducted exploratory analyses using generalized linear models to assess whether having sibling pairs in the sample had any impact on statistical independence in our results.

## Results

### Sample characteristics of the caregivers

A total of 485 caregivers, taking care of 558 adolescents, participated in this phase of the study (see Table 1). The sample of adolescents comprised of 75 sibling pairs, hence the mismatch with the number of caregivers. The mean (range) age of the caregivers across the sample was 43.5 (13–78) years. The majority of these caregivers were biological mothers of the care recipients (343; 71.61%). Close to half of the caregivers across the sample were living with HIV (228; 47%), a majority (415; 85.6%) were females, about two thirds (321; 66.2%) were married and living with their spouses, three quarters (364; 75.1%) were Christians while a majority (420; 86.6%) had up to a primary level of education. A substantial proportion of the caregivers (193; 39.8%) depended on seasonal subsistence farming for their livelihood, and a similar proportion (211; 43.5%) were small scale traders. Groupwise, caregivers of PHI adolescents were more likely to be non-biological parents, have a higher body mass index (BMI), and be in professional employment compared to caregivers of both PHEU and HUU adolescents. Moreover, caregivers of PHI and PHEU adolescents were more likely to be older, seropositive, and living single compared to caregivers of HUU adolescents. We did not find differences in sex and socioeconomic status among the different caregiver groups. Table 1 gives a detailed description of the sociodemographic characteristics and other related variables of the caregivers with their statistical tests of difference across the three groups of caregivers.

**Table 1:** Sample characteristics of primary caregivers of perinatally HIV infected, perinatally HIV exposed but uninfected and HIV unexposed/uninfected adolescents *(N* = *485)*

Sample characteristic	Caregivers of adolescents living with HIV (n = 195)	Caregivers of adolescents perinatally exposed to HIV but uninfected (n = 128)	Caregivers of HIV unexposed/ uninfected adolescents (n = 162)	Whole sample (n = 485)	Statistical difference
	Mean (SD) or Median (IQR) or n %	Mean (SD) or Median (IQR) or n %	Mean (SD) or Median (IQR) or n %	Mean (SD) or Median (IQR) or n (%)	
*Sex of caregiver*					
Female	168 (86.2%)	115 (89.8%)	132 (81.5%)	415 (85.6%)	0.1
Caregivers mean age (years) **[OM = 2]**	44.6 (12.1)	44.8 (10.3)	41.1 (10.1)	43.5 (11.1)	0.002
*Caregiver's level of education*					
None	51 (26.2%)	40 (31.3%)	68 (42%)	159 (32.8%)	
Primary level	101 (51.8%)	78 (60.9%)	82 (50.6%)	261 (53.8%)	< 0.001
Secondary level	28 (14.4%)	10 (7.8%)	10 (6.2%)	48 (9.9%)	
Tertiary level	15 (7.7%)	00 (0%)	2 (1.2%)	17 (3.51%)	
*Caregiver's marital status*					
Never married	17 (8.7%)	4 (3.1%)	1 (0.6%)	22 (4.5%)	
Married	112 (57.4%)	67 (52.3%)	142 (87. %7)	321 (66.2%)	< 0.001
Widowed/divorced	66 (33.9%)	57 (44.5%)	19 (11.7%)	142 (29.3%)	
*Caregiver's occupation*					
Farmer	66 (33.9%)	45 (35.2%)	82 (50.6%)	193 (39.8%)	
Small scale trader	86 (44.1%)	62 (48.4%)	63 (38.9%)	211 (43.5%)	
Casual labourer	29 (14.9%)	21 (16.4%)	13 (8%)	63 (13%)	< 0.001
Professional	14 (7.2%)	0 (00%)	4 (2.5%)	18 (3.7%)	
*Caregiver's religion*					
Christianity	155 (79.5%)	97 (75.8%)	112 (69.1%)	364 (75.1%)	
Islam	25 (12.8%)	21 (16.4%)	18 (11.1%)	64 (13.2%)	0.01
Traditional	15 (7.7%)	10 (7.8%)	32 (19.8%)	57 (11.7%)	
*Caregiver's HIV status*					
Seronegative	67 (34.4%)	30 (23.4%)	160 (98.8%)	257 (53.0%)	< 0.001
Seropositive	128 (65.6%)	98 (76.6%)	02 (1.2%)	228 (47.0%)	
Caregiver’s mean body mass index (BMI)	24.2 (6.3)	21.9 (4.7)	22.9 (4.0)	23.2 (5.3)	< 0.001
Caregiver’s mean socioeconomic status score	2.0 (1.8)	1.5 (1.3)	1.5 (1.3)	1.7 (1.6)	0.08
Caregiver’s median depressive symptoms	7.0 (4.0–9.0)	6.0 (3.5–9.0)	5.0 (3.0–8.0)	6.0 (3.0–8.0)	0.07
Caregiver’s median parenting stress score	64.0 (57.0–71.0)	60.0 (55.0–68.0)	65.0 (59.0–71.0)	64.0 (56.0–70.0)	0.03

% are column percentages

SD, standard deviation; OM, observations missing

### Sample characteristics of adolescents receiving care

Slightly more than half (296; 53.1%) of the adolescents across the sample were female, with a mean (range) age of 13.7 (11–17) years. Groupwise, PHEU adolescents were more likely to be younger compared to both HUU and PHI adolescents. On average, the adolescents had spent 5.4 years in school across the sample. Both PHI and PHEU adolescents were more likely to be orphans, compared to HUU adolescents. Besides, PHI adolescents were more likely to present with lower mid-upper arm circumference (MUAC) and BMI (see Table 2).

**Table 2:** Sample characteristics of the adolescents receiving care *(N* = *558)*

Sample characteristic	Adolescents living with HIV (n = 201)	Adolescents perinatally exposed to HIV but uninfected (n = 157)	HIV unexposed/ uninfected adolescents (n = 200)	Whole sample (n = 558)	Statistical difference
	Mean (SD) or n %	Mean (SD) or n %	Mean (SD) or n %	Mean (SD) or n (%)	
*Relationship with caregiver ****[OM* = *5]***					
Biological mother	111 (56.4%)	124 (78.5%)	163 (82.3%)	398 (72.0%)	
Biological father	21 (10.7%)	12 (7.6%)	28 (14.1%)	61 (11.0%)	< 0.001
Grandparent	23 (11.7%)	13 (8.2%)	00 (0.0%)	36 (6.5%)	
Another relative	33 (16.8%)	8 (5.1%)	6 (3.0%)	47 (8.5%)	
Sibling	9 (4.6%)	1 (0.6%)	1 (0.5%)	11 (2.0%)	
Mean age (years)	13.8 (1.5)	13.3 (1.4)	14.1 (1.6)	13.7 (1.6)	< 0.001
*Adolescent sex*					
Female	104 (51.7%)	88 (56.1) %	104 (52.0%)	296 (53.1%)	0.7
Mean number of schooling years **[OM = 14]**	5.3 (2.2)	5.3 (2.1)	5.6 (1.6)	5.4 (2)	0.3
*Grade retention ****[OM* = *4]***					
No	106 (53.3%)	102 (65.4%)	103 (51.8%)	311 (56.1%)	0.02
Yes	93 (46.7%)	54 (34.6%)	96 (48.2%)	243 (43.9%)	
*Orphan status*					
Both parents alive	98 (48.5%)	96 (60.8%)	181 (91.4%)	375 (67.2%)	
One parent alive	67 (33.2%)	57 (36.1%)	17 (8.6%)	141 (25.3) %	< 0.001
Both parents deceased	37 (18.3%)	05 (3.2%)	00 (0.0%)	42 (7.5%)	
Mean mid upper arm circumference (MUAC) **[OM = 20]**	20.5 (2.9)	21.1 (2.6)	21.5 (2.6)	21.1 (2.8)	0.001
Mean body mass index (BMI) **[OM = 18]**	17.3 (2.8)	17.5 (2.5)	17.7 (2.2)	17.5 (2.5)	0.04
Mean head circumference **[OM = 18]**	53.2 (1.6)	53.2 (1.6)	53.3 (1.7)	53.3 (1.6)	0.03

% are column percentages

SD, standard deviation; OM, observations missing

### Health-related quality of life profile of caregivers

Caregivers of HUU children had significantly higher HRQoL scores than the other caregivers in the overall HRQoL domain. For all the HRQoL subscales (except energy/fatigue and social functioning domains), caregivers of HUU adolescents had significantly higher mean HRQoL scores compared to caregivers of both PHEU and PHI adolescents (see Table 3). Caregivers of PHI and PHEU adolescents had similar mean overall HRQoL scores. Additionally, caregivers of PHI adolescents reported significantly lower mean HRQoL scores across all subscales (except for physical functioning, emotional wellbeing and general health domains) than caregivers of PHEU adolescents. Across the sample, the largest mean quality of life deficits was observed in energy/fatigue (− 46.9%), pain (− 36.3%), social functioning (− 34.7%) and emotional wellbeing (− 32.7%). Table 3 summarizes a break-down of these results, and Additional file 4 gives a graphical distribution of the mean HRQoL across the caregivers.

**Table 3:** Health-related quality of life profile among the caregivers (*N* = *485*)

Variable	Caregivers of adolescents living with HIV (n = 195)		Caregivers of adolescents perinatally exposed to HIV but uninfected (n = 128)		Caregivers of HIV unexposed/ uninfected adolescents (n = 162)		Whole sample (n = 485)		P value
	Mean (SD)	Median (IQR)	Mean (SD)	Median (IQR)	Mean (SD)	Median (IQR)	Mean (SD)	Median (IQR)	
Overall HRQoL	73.0 (15.6)	77.1 (63.5 –84.4)	72.9 (15.9)	78.1 (65.0–85.3)	78.9 (11.4)	81.8 (72.6–87.2)	75.0 (14.7)	79.2 (67.1–85.8)	< 0.001
Physical functioning	90.7 (21.2)	100.0 (100.0–100.0)	89.7 (22.6)	100.0 (92.5–100.0)	97.6 (8.3)	100.0 (100.0–100.0)	92.7 (18.7)	100.0 (100.0–100.0)	0.02
Role limitation due to physical health	70.8 (36.8)	100.0 (50.0–100.0)	76.0 (35.7)	100.0 (50.0–100.0)	81.2 (31.5)	100.0 (50.0–100.0)	75.6 (35.1)	100.0 (50.0–100.0)	0.05
Role limitation due to emotional problems	68.2 (37.7)	100.0 (33.3–100.0)	69.0 (39.7)	100.0 (33.3–100.0)	82.5 (31.8)	100.0 (66.7–100.0)	73.2 (36.9)	100.0 (33.3–100.0)	0.003
Vitality (Energy/fatigue)	51.7 (16.6)	50.0 (40.0–65.0)	54.4 (17.3)	50.0 (45.0–65.0)	53.8 (14.4)	50.0 (40.0–65.0)	53.1 (16.1)	50.0 (40.0–65.0)	0.2
Emotional well-being	68.5 (17.7)	72.0 (56.0–84.0)	63.6 (17.9)	60.0 (48.0–80.0)	68.7 (18.4)	72.0 (48.0–84.0)	67.3 (18.1)	68.0 (52.0–84.0)	0.03
Social functioning	64.9 (20.0)	62.5 (50.0–75.0)	65.9 (19.9)	62.5 (50.0–75.0)	65.1 (20.2)	62.5 (50.0–75.0)	65.3 (20.0)	62.5 (50.0–75.0)	0.8
Pain	60.7 (24.5)	55.0 (45.0–77.5)	63.1 (24.2)	61.3 (45.0–77.5)	67.7 (23.6)	67.5 (45.0–90.0)	63.7 (24.3)	65.0 (45.0–77.5)	0.04
General health	75.1 (17.7)	80.0 (65.0–90.0)	74.0 (17.6)	77.5 (62.5–90)	83.0 (12.2)	90.0 (80.0–90.0)	77.4 (16.5)	85.0 (70.0–90.0)	< 0.001

HRQoL, health related quality of life; SD, standard deviation

### Correlates of HRQoL among primary caregivers

In the univariable linear regression analyses across the sample, all variables except sex and grade retention of adolescents receiving care were significantly associated with the subscale and overall HRQoL scores of the caregivers (at p < 0.05). Apart from grade retention, the other variables met the set a priori significance level of (p < 0.20) in some of the HRQoL subscales for consideration in the multivariable linear regression analyses. Details of the univariable analyses across the sample are presented in Additional file 1.

We also conducted univariable linear regression analyses between the independent variables and the quality-of-life profiles (overall and sub-domains) for each study group. Details of these results are presented in Additional file 2.

In the multivariable linear regression analyses, increasing depressive symptoms among caregivers was strongly associated with declining overall (*β* =  − *2.00, 95% CI* − *2.3,* − *1.7; p* < *0.001*) and subscale HRQoL (Table 4). Likewise, increasing age of caregivers was significantly associated with decreasing overall (*β* =  − *0.2, 95% CI* − *0.3,* − *0.1; p* < *0.01*) and subscale (physical functioning, role limitations due to physical health and social functioning) HRQoL (Table 4). Similarly, caring for a PHEU adolescent was significantly associated with declining overall (*β* =  − *3.7, 95% CI* − *7.3,* − *0.2; p* < *0.05*) and subscale (general health) HRQoL. This was also the case for a caregiver taking care of a PHI adolescent which was associated with a reducing overall (*β* =  − *4.8, 95% CI* − *8.0,* − *1.5; p* < *0.01*) and subscale (role limitations due to emotional problems, energy/fatigue and general health) HRQoL (Table 4). On the other hand, having a professional job relative to small scale subsistence farming was strongly associated with improved overall HRQoL (*β* = *8.6, 95% CI 2.5, 14.7; p* < *0.01*). We did not find evidence of an association between caregivers' HIV status and HRQoL both at the overall and subscale levels.

**Table 4:** Multivariable linear regression analysis of correlates of HRQoL among caregivers

	β-coefficient (95% CI) of HRQoL domains and overall scale as dependent variables								
Independent variables	Overall HRQoL	Physical functioning	Role limitations due to physical health	Role limitations due to emotional problems	Energy/fatigue	Emotional wellbeing	Social functioning	Pain	General health
Adolescent's age								− 1.6** (− 3.0; − 0.3)	
*Adolescent's sex*									
Female					Ref				
Male					− 2.9** (− 5.5; − 0.3)				
Adolescent's head circumference					0.9* (− 0.1; 1.7)				
*Adolescent's orphan status*									
Not one									Ref
Partial orphan									1.0 (− 2.4; 4.4)
Full orphan									5.97* (− 0.01; 12.0)
*Adolescent's HIV exposure*									
Unexposed uninfected	Ref	Ref	Ref	Ref	Ref	Ref	Ref	Ref	Ref
Exposed-uninfected	− 3.7** (− 7.3; − 0.2)	− 5.0* (− 10.8; 0.8)	0.4 (− 9.1; 9.9)	− 8.9* (− 18.6; 0.8)	− 1.5 (− 6.3; 3.3)	− 3.3* (− 7.8; 1.23)	3.3 (− 2.3; 9.0)	− 0.1 (− 7.8; 7.7)	− 5.8** (− 10.6; − 1.0)
Living with HIV	− 4.8*** (− 8.0; − 1.5)	− 3.1 (− 8.9; 2.7)	− 6.8* (− 15.4; 1.8)	− 11.5** (− 20.3; − 2.7)	− 4.8** (− 9.6; − 0.1)	0.2 (− 3.9; 4.3)	1.6 (− 3.5; 6.8)	− 2.0 (− 9.7; 5.6)	− 6.6*** (− 11.3; − 1.9)
*Caregiver's HIV status*									
Seronegative	Ref	Ref	Ref	Ref	Ref	Ref	Ref	Ref	Ref
Seropositive	− 0.04 (− 2.9; 2.8)	0.6 (− 4.7; 5.9)	− 1.8 (− 9.4; 5.9)	− 1.5* (− 9.3; 6.3)	− 3.0* (− 1.4; 7.3)	0.1 (− 3.6; 3.7)	− 0.6 (− 5.1; − 4.0)	− 3.9 (− 10.8; 3.0)	− 2.0 (− 5.9; 2.0)
*Adolescent's relationship with caregiver*									
Biological mother		Ref			Ref			Ref	
Biological father		3.1 (− 2.8; 9.1)			0.8 (− 3.8; 5.3)			− 1.2 (− 8.3; 5.9)	
Grandparent		− 15.6*** (− 24.1; − 7.6)			3.0 (− 3.2; 9.1)			− 12.1** (− 21.9; − 2.4)	
Other relative		− 1.8 (− 8.2; 4.6)			6.0** (0.7; 11.3)			− 2.4 (− 10.7; 5.9)	
Sibling		5.2 (− 6.6; 17.0)			10.7** (1.1; 20.3)			− 1.6 (− 16.4; 1.)	
*Caregiver's religion*									
Christianity								Ref	Ref
Islam								5.4* (**− **11.5; 0.6)	− 3.14* (− 7.24; 0.95)
Traditional								4.3 (− 2.2; 10.7)	4.4* (0.01; 8.9)
*Caregiver's educational level*									
No formal education		Ref		Ref		Ref	Ref		Ref
Primary		− 1.2 (− 4.9; 2.5)		− 1.4 (− 8.3; 5.4)		− 0.4 (− 3.7; 2.8)	− 1.4 (− 5.3; 2.6)		− 2.8 (− 5.9; 0.4)
Secondary		− 7.3** (− 13.4; − 1.2)		− 9.9* (− 21.2; 1.5)		3.8* (− 1.7; 9.2)	3.9 (− 2.6;10.4)		− 1.9 (− 7.2; 3.3)
Tertiary		− 3.6 (− 14.5; 7.3)		13.0* (− 5.6; 31.5)		5.0 (− 4.8; 14.7)	4.5 (− 6.1; 15.1)		5.8* (− 2.2; 13.7)
Caregiver's BMI			0.6* (0.1; 1.2)						
Caregiver’s depressive symptoms (PHQ-9 scores)	− 2.0*** (− 2.3; − 1.7)	− 1.2*** (− 1.6; − 0.7)	− 3.8*** (− 4.6; − 4.0)	− 4.0*** (− 4.9; − 3.2)	− 1.5*** (− 1.8; − 1.1)	− 2.4*** (− 2.8; − 2.0)	− 1.8*** (− 2.3; − 1.3)	− 2.4*** (− 2.9; − 1.8)	− 1.3*** (− 1.7; − 1.0)
Caregiver’s socioeconomic score	0.5 (− 0.2; 1.3)	− 0.2 (− 1.3; 1.0)	0.5 (− 1.5; 2.5)	2.3** (0.1; 4.4)	0.5 (− 0.5; 1.4)	0.9* (− 0.1; 2.0)	− 0.1 (− 1.4; 1.1)		
Caregiver's parenting stress (PSS scores)					− 0.3*** (− 0.4; − 0.1)				
Caregiver’s age (years)	− 0.2*** (− 0.3; − 0.1)	− 0.2*** (− 0.4; − 0.04)	− 0.3** (− 0.6; − 0.04)				− 0.2** (− 0.3;0.0)		
*Caregiver's occupation*									
Subsistence farmer	Ref							Ref	
Small scale trader	0.2 (− 2.2; 2.6)					− 2.4* (− 5.6; 0.8)		2.4 (− 2.1; 6.9)	
Casual labourer	1.8 (− 1.7; 5.3)					3.8* (− 0.7; 8.3)		6.8** (0.2; 13.3)	
Professional	8.6*** (2.5; 14.7)					2.9 (− 6.0; 11.7)		6.2 (− 6.8; 19.2)	
n of the final model	483	477	480	485	461	485	483	477	484
R-squared for the final model	36.3%	19.6%	21.1%	21.9%	24.4%	29.7%	13.3%	19.4%	17.4%

Bolded—some level of significance

HRQoL, health-related quality of life; BMI, Body Mass Index

^*^p < 0.20; **p < 0.05; ***p < 0.01

A few other variables were also associated with an increase or a decrease of HRQoL at the subscale level. Being a grandparent relative to a biological mother among caregivers was significantly associated with reduced HRQoL in the physical functioning domain (*β* =  − *15.6, 95% CI* − *24.1,* − *7.6; p* < *0.001*) and pain domain (*β* =  − *12.1, 95% CI* − *21.9,* − *2.4; p* < *0.05*) (Table 4). Likewise, a unit increase of an adolescent age was significantly associated with a 1.6-point reduction in quality-of-life scores in the pain domain. Also, caring for a male adolescent relative to a female one was associated with a reduction of HRQoL in the vitality (energy/fatigue) domain (*β* =  − *2.9, 95% CI* − *5.5,* − *0.3; p* < *0.05*). Furthermore, a unit increase in parenting stress among caregivers was associated with a 0.3-point reduction in quality-of-life scores in the vitality (energy/fatigue) domain. Besides, having higher educational achievement was associated with reduced HRQoL scores in the physical functioning domain. A unit increase in caregiver's socioeconomic status was associated with a 2.3-point improvement in HRQoL in the role limitations due to emotional problems domain. Our exploratory analyses assessing for the presence of statistical independence in our data (due to the presence of sibling pairs) did not find evidence for statistical independence.

## Discussion

Optimal caregiving is essential for adolescents to thrive. In the current study, we set out to document the HRQoL status of primary caregivers taking care of adolescents perinatally exposed to HIV in a rural area of coastal Kenya. Our results show that in the current era of highly active ART, most caregivers of adolescents perinatally exposed to HIV are biological parents, often their mothers, unlike in the earlier years of the epidemic where many were ageing grandmothers [6, 7]. Overall, the HRQoL of caregivers in this sample was within the average level (above 50%). However, our sample presented large HRQoL deficits in vitality (energy/fatigue), pain, social functioning, and emotional wellbeing domains. These domains highlight the important HRQoL targets that can be plausibly prioritized for interventions seeking to improve the quality of life of caregivers of adolescents perinatally exposed to HIV in the study setting. A closer examination of these domains indicates the critical need for addressing the mental and psychosocial needs of these caregivers.

There is a large body of evidence showing that adults living with HIV are generally at risk of poorer quality of life outcomes than demographically-comparable HIV uninfected counterparts partly because of social circumstances, relationship issues, comorbidities, and stigma in addition to their underlying infection [46, 47]. However, few studies have examined the effect of caregivers' and care recipients’ HIV serostatus on the carer's HRQoL in SSA. In our study, only the HIV status of the care recipients predicted HRQoL among the caregivers in adjusted models. Contrary to our initial hypothesis, caregivers' HIV serostatus was not associated with HRQoL. This finding could perhaps be an indication that the distress arising from caring for an adolescent living with HIV has more negative impact than the carer's own status on the HRQoL. Indeed, a recent qualitative exploration of the challenges faced by adolescents living with HIV in the same study setting identified several challenges that the adolescents face including poverty, poor mental and physical health, unsupportive school system, high levels of stigma, problems with HIV disclosure, and medication adherence [48]. Arguably, such challenges could be taking a toll on the caregivers HRQoL; however, more studies are required to understand the role of these factors on the caregivers' HRQoL. Our observation could be further explained by the significant economic burden that caregivers of adolescents living with HIV are likely to face in their caregiving roles. Indeed, a recent cross-sectional study in the same study setting documented significant economic burden (especially for transportation and medication costs) among caregivers of perinatally HIV infected adolescents [49]. However, it could be that some of the adult carers are HIV uninfected and yet taking care of adolescents living with HIV or even vice-versa whereby a carer living with HIV is taking care of PHEU adolescent who poses fewer challenges, he/she (adolescent) is disease-free. This discrepancy of HIV serostatus could have limited the effects on the overall HRQoL outcomes.

Another key finding from the present study was the predictive effect of depressive symptomatology on HRQoL (overall and all subscales) among caregivers. Our finding on worsening HRQoL with an increasing burden of depression is consistent with the results of a recent cross-sectional study which examined the impact of cognitive and mental health outcomes on the HRQoL of a low literacy adult population in the same study setting [42]. Besides, there have been several reports of poor psychosocial and mental health outcomes in both HIV exposed and HIV unexposed individuals in our study setting [42, 48, 50–52]. High rates of untreated depressive symptoms, perhaps because of HIV-related stigma and difficulties in accessing care, could explain why it is such a strong predictor of HRQoL in this sample [52]. Untreated depression could result in impaired economic productivity, reduced ability to perform work and social roles, loss of relationships, physical decline, and problem-solving deficits for the caregivers, hence negatively impacting their HRQoL [53].

Among caregivers' sociodemographic factors, only socioeconomic status correlated positively with HRQoL while age, education level, and relationship with adolescents influenced HRQoL negatively. Similar to findings from other developing countries, having a professional job relative to subsistence farmers (many of whom are likely to be unemployed) was associated with improved overall HRQoL [54]. Arguably, individuals in formal employment are more likely to have higher educational level, enjoy higher wages and better working conditions and work environments compared to those without professional employment, thus less likely impacted by high caregiving burden (as may be the case for carers of adolescents living with HIV [49]), hence the improvement in their HRQoL. Better socioeconomic status has long been associated with good health outcomes in the literature [15]. Individuals of higher socioeconomic status are likely to have a more enlightened attitude towards disease management, better health-seeking behaviours, and more likely to be employed hence easily access healthcare services, which could invariably enhance their HRQoL. In our study, a unit increase in socioeconomic status score was associated with 2.3-point improvement in HRQoL in the role limitations due to emotional problems domain.

We also found out that having secondary level education relative to lack of formal education was strongly associated with reducing HRQoL in the physical functioning domain. The reason for this observation is not well understood; more detailed studies (with larger samples) are needed to explore the impact of educational status on HRQoL. We also noted that a unit increase in caregivers age was associated with declining overall, and subscale (physical functioning, role limitations due to physical health and social functioning) HRQoL. A study in Namibia did not find any association between age and HRQoL [19]. However, this study had a smaller sample size (n = 97), composed of general caregivers (not of HIV exposed adolescents), did not adjust for the caregivers' HIV status, and most of them had a chronic illness that prevented them from working. Our finding could potentially be due to the direct effect of ageing on caregivers (reduced muscle mass) which has been linked with lowered muscle strength, reduced maximal aerobic exercises, and decreased bone density [15, 55]. It could also be related to HIV treatment factors such as adherence, viral suppression, among others. However, these factors were not measured in the current study. Moreover, cumulative exposure to risk factors, including sedentary lifestyles, loneliness usually associated with reduced physical activity could also play a role in our observation. Related to this finding, we also observed that being a grandparent relative to a biological mother was associated with a reduced quality of life in the physical functioning and pain domains. Older caregivers (many of whom are grandparents) are likely to experience an excess risk of caregiving burden, child behavioural difficulty, weakened support systems, geriatric syndromes (e.g. impaired mobility, somatic symptoms such as chronic pain) which are likely to harm their health outcomes [56, 57]. In Uganda, however, older caregivers of HIV exposed people were more likely to report better health compared to those without caregiving responsibility [12].

Among child characteristics, we found out that caring for a male adolescent influenced caregivers HRQoL negatively. Likewise, the increasing age of adolescent was associated with a decline in caregivers HRQoL in the pain domain. In this cohort, both male sex and increasing age are risk factors for poor adolescent health outcomes such as multiple health risk behaviour [33]. Thus, it is plausible that the care for adolescent males involves various sources of distress for the carers, and these have implications for the carers HRQoL. Also, this period coincides with multiple adjustments for the growing adolescent and the caregiver such as disclosing the HIV status to the adolescent, handling sexuality issues for the adolescent (e.g. forming relationships) and how to manage the potentially elevated risk of HIV-associated psychosocial problems. Such adjustments could partly explain the decline in caregivers HRQoL, especially in the absence of adequate psychosocial support to the caregivers on coping positively [58, 59]. This could also be partly explained by an increasing caregiving burden in the caregiver as the child grows. It may translate into more needs for adolescent in terms of food, educational requirements such as school fees, and active parenting issues (such as disciplining issues) and others. Nonetheless, we cannot make firm conclusions regarding the role of these factors in the current analyses; more research is needed to explore them further, particularly within the context of qualitative studies.

### Strengths and limitations

To our knowledge, this is among the few studies in SSA that have documented the quality of life of caregivers of adolescents perinatally exposed to HIV in the present era of effective ART. The study has few strengths. First is has a relatively large sample size. Secondly, the inclusion of two comparison groups; caregivers of PHEU and caregivers of HUU adolescents enabled us to adequately compare HRQoL outcomes across the 3 groups of caregivers in the same setting. Our study utilized self-report data collection measures, e.g., the HRQoL questionnaire. The reliance on subjective reporting may have led to under-reporting or over-reporting of outcomes; however, this was minimized by training the administrators. This bias was also minimized by adequately adapting the self-report measures, yielding good psychometric properties. Caregivers of HIV unexposed and uninfected adolescents were not directly tested for HIV and may have introduced some selection bias. However, their recruitment was limited to caregivers who willingly shared their HIV test results at the time of pregnancy with the adolescent. Additionally, a brief screening checklist was utilized to exclude caregivers who were experiencing severe illness or were having recurring health problems to minimize the possibility of including caregivers who were potentially HIV-infected in this group. We also experienced few cases of non-response (this was mainly attributed to silent refusal or direct refusal of further contact with research team). However, the non-respondents did not differ by HIV group composition and sex distribution. Furthermore, our study precludes any conclusions on causality in the observed associations given its cross-sectional design. Also, our study did not include other potentially important variables when examining the caregivers’ HRQoL such as the mental health outcomes and quality of life of the care recipients, which may have given us a comprehensive picture of the present findings.

## Conclusions

The results of the present study draw attention to crucial implications for healthcare providers and policymakers. First, primary caregivers, especially those taking care of adolescents perinatally exposed to HIV at the Kenyan Coast are vulnerable regardless of their own HIV serostatus. This highlights the crucial need for inclusive and multi-component interventions tailored to the caregivers' physical, psychosocial, and mental health aspects to meaningfully enhance their quality of life and that of their adolescents. Family-based programmes addressing, for instance, household psychosocial risk factors would be beneficial in this setting. Secondly, it is worthwhile to focus more intensive support to caregivers with an elevated risk of mental health problems, those who are ageing, those living in the poorest households, and those with poorer health states. Properly designed epidemiological/longitudinal studies are urgently needed to understand the underlying mechanisms and longer-term implications of the correlates of caregivers HRQoL identified in the present study.

## Supplementary Information

**Additional file 1: Table S1**. Univariable linear regression analysis of correlates of HRQoL among the primary caregivers.

**Additional file 2: Table S2**. Univariable linear regression analysis of the correlates of HRQoL among primary caregivers of adolescents living with HIV. **Table S3**. Univariable linear regression analysis of correlates of HRQoL among primary caregivers of adolescents perinatally exposed to HIV but uninfected. **Table S4**. Univariable linear regression analysis of the correlates of HRQoL among primary caregivers of HIV unexposed and uninfected adolescents.

**Additional file 3: Table S5**. Description of the HRQoL measure (RAND SF-36).

**Additional file 4**. Graphical distribution of the mean HRQoL scores across the three groups of caregivers.

## Data Availability

No additional data are available. Anyone interested in accessing the data reported in this article is free to write to the Data Governance Committee of the KEMRI Wellcome Trust Research Programme who will review the application and advise as appropriate and ensure that uses are compatible with the consent obtained from participants for data collection. Requests can be sent to the coordinator of the Data Governance Committee using the following email: dgc@kemri-wellcome.org.

## Abbreviations

AHOS: Adolescent Health Outcomes Study
ANOVA: Analysis of variance
BMI: Body mass index
CI: Confidence interval
CGMRC: Centre for Geographic Medicine Research, Coast
HIV: Human immunodeficiency virus
HRQoL: Health-related quality of life
HUU: HIV unexposed uninfected
IQR: Interquartile range
KEMRI: Kenya Medical Research Institute
KHDSS: Kilifi Health and Demographic Surveillance System
MUAC: Mid-upper arm circumference
OM: Observations missing
PHEU: Perinatally HIV exposed but uninfected
PHI: Perinatally HIV infected
PHQ-9: 9-Item Patient Health Questionnaire
PSS: Parental Stress Scale
RAND-SF 36: RAND 36-Item Short Form Survey questionnaire
SD: Standard deviation
SERU: Scientific Ethics Review Unit
SSA: Sub-Saharan Africa
WHO: World Health Organization
