# Supplementary material for: Correlates of health-related quality of life in primary caregivers of perinatally HIV infected and HIV exposed uninfected adolescents at the Kenyan Coast
Source: Health Qual Life Outcomes. 2022 Jan 21;20:11. doi: 10.1186/s12955-022-01915-z (PMC8780308; doi:10.1186/s12955-022-01915-z)
Supplement: Supplementary file 1 — Additional file 1: Table S1. Univariable linear regression analysis of correlates of HRQoL among the primary caregivers. [file 12955_2022_1915_MOESM1_ESM.docx]

**Supplementary Table 1. Univariable linear regression analysis of correlates of HRQoL among the primary caregivers**

|  | **β-coefficient (95% CI) of HRQoL domains and overall scale as dependent variables** | | | | | | | | | |
| --- | --- | --- | --- | --- | --- | --- | --- | --- | --- | --- |
| Independent variables | Overall HRQoL | Physical functioning | Role Limitations due to physical health | | Role Limitations due to emotional problems | Energy/ fatigue | Emotional wellbeing | Social functioning | Pain | General health |
| Participating adolescent’s age [OM=2] | 0.3 (−0.5;1.2) | −0.1  (−1.2; 1.0) | 0.3  (−1.8; 2.4) | | 0.9  (−1.3; 3.1) | **1.0****  (0.02; 1.9) | **0.8***  (−0.3; 1.9) | 0.3  (−0.9; 1.5) | **−1.1***  (−2.5; 0.3) | 0.5  (−0.5; 1.5) |
| Participating adolescent's sex [OM=2] |  |  |  | |  |  |  |  |  |  |
| Female | Ref | Ref | Ref | | Ref | Ref | Ref | Ref | Ref | Ref |
| Male | −1.4 (−4.1;1.2) | −**2.2***  (−5.6; 1.1) | −3.3  (−9.5; 3.0) | | −2.5  (−9.1; 4.2) | **−2.6***  (−5.4; 0.3) | 1.2  (−2.0; 4.5) | −0.1  (−3.6; 3.5) | 0.04 (−4.3; 4.4) | −0.7 (−3.7; 2.3) |
| Participating adolescent’s MUAC [OM=20] | 0.2  (−0.3; 0.7) | 0.2  (−0.4; 0.9) | 0.8  (−0.4; 1.9) | | 0.4  (−0.8; 1.6) | **0.6****  (0.03; 1.1) | −0.3  (−0.9; 0.3) | −0.2  (−0.8; 0.5) | −0.3  (−1.1; 0.5) | 0.3  (−0.2; 0.9) |
| Participating adolescent's head circumference  [OM=18] | **1.0****  (0.1; 1.8) | 0.6  (−0.5; 1.6) | 0.5  (−1.4; 2.5) | | **2.3****  (0.2; 4.3) | **1.5*****  (0.6; 2.3) | **0.7***  (−0.3; 1.7) | **1.1*****  (0.04; 2.2) | 1.1  (−0.3; 2.4) | **1.0****  (0.04; 1.9) |
| Participating adolescent’s number of years in school [OM=8] | **0.5***  (−0.1; 1.2) | −0.1  (−0.9; 0.8) | 1.2  (−0.4; 2.8) | | 1.2  (−0.5; 2.9) | **1.1*****  (0.4; 1.8) | **0.6***  (−0.2; 1.5) | **1.1***  (0.2; 2.0) | 0.5  (−0.6; 1.7) | 0.1  (−0.7; 0.9) |
| Participating adolescent's HIV Exposure |  |  |  | |  |  |  |  |  |  |
| Unexposed uninfected | Ref | Ref | Ref | | Ref | Ref | Ref | Ref | Ref | Ref |
| Exposed Uninfected | **−6.0***** (−9.3; -2.6) | **−7.9***** (−12.2;−3.6) | −5.2 (−13.3; 2.9) | | **−13.5***** (−22.0;−5.0) | 0.6 (−3.1; 4.4) | **−5.0**** (−9.2; −0.9) | 0.8 (−3.9; 5.4) | −**4.6***  (−10.2; 1.0) | −**9.0***** (−12.8; −5.3) |
| HIV Infected | **−6.0***** (−9.0;−2.9) | **−6.9*****  (−10.7;−3.0) | **−10.4*****  (−17.7;−3.1) | | **−14.3*****  (−21.9;−6.7) | −2.1  (−5.4;−1.3) | −0.2  (−3.9;3.6) | −0.2  (−4.4;4.0) | **−7.0*****  (−12.1;−2.0) | **−8.0*****  (−11.3;−4.6) |
| Grade retention by Participating adolescent [OM=6] |  |  |  | |  |  |  |  |  |  |
| No | Ref | Ref | Ref | | Ref | Ref | Ref | Ref | Ref | Ref |
| Yes | −0.1  (−2.7; 2.6) | −0.3  (−3.7; 3.2) | −0.8  (−7.1; 5.6) | | −1.3  (−8.0; 5.4) | 0.1  (−2.8; 3.0) | 0.7  (−2.6; 4.0) | 0.9  (−2.7; 4.5) | −1.7  (−6.1; 2.8) | 0.8  (−2.2; 3.8) |
| **Supplementary Table 1 continued** | | | | | | | | | | |
| **Independent variables** | **Overall HRQoL** | **Physical functioning** | **Role Limitations due to physical health** | | **Role Limitations due to emotional problems** | **Energy/ fatigue** | **Emotional wellbeing** | **Social functioning** | **Pain** | **General health** |
| Participating adolescent's orphan status [OM=1] |  |  |  | |  |  |  |  |  |  |
| Both parents alive | Ref | Ref | Ref | | Ref | Ref | Ref | Ref | Ref | Ref |
| One parent alive | **−4.5***** (−7.6;−1.5) | **−4.3**** (−8.1;−0.5) | **−8.0**** (−15.2;−0.7) | | **−8.8**** (−16.4;−1.2) | **−2.7*** (−6.0;−0.7) | **−2.9*** (−6.7; 0.8) | **−3.0**** (−7.2; −1.1) | **−4.8*** (−9.8; 0.2) | −**3.5****  (−6.9;−0.1) |
| Both parents deceased | **−7.2***** (−12.0;−2.4) | **−14.5***** (−20.6;−8.4) | **−14.0**** (−25.6; −2.4) | | **−14.1**** (−26.4; −1.9) | 0.8 (−4.6; 6.1) | 1.6 (−4.4; 7.6) | **−8.4**** (−15.0;−1.8) | **−11.2***** (−19.2;−3.2) | 2.4  (−3.1; 7.9) |
| Caregiver HIV Status |  |  |  | |  |  |  |  |  |  |
| Seronegative | Ref | Ref | Ref | | Ref | Ref | Ref | Ref | Ref | Ref |
| Seropositive | **−3.6***** (−6.3;−1.0) | −**2.3*** (−5.6; 1.1) | **−5.9***** (−12.2; −0.3) | | **−9.8*****  (−16.4; −3.3) | −1.1 (−4.0; 1.8) | −1.7 (−4.9; 1.6) | 0.2 (−3.4; 3.8) | **−3.9***  (−8.2; 0.4) | **−7.2*****  (−10.1;−4.4) |
| Caregiver sex |  |  |  | |  |  |  |  |  |  |
| Female | Ref | Ref | Ref | | Ref | Ref | Ref | Ref | Ref | Ref |
| Male | 2.2  (−1.5; 5.9) | −0.5  (−5.2; 4.3) | 4.7  (−4.2; 13.6) | | **10.2****  (0.8; 19.5) | 0.9  (−3.2; 5.0) | 2.7  (−1.9; 7.3) | **3.7***  (−1.4; 8.7) | 2.7  (−3.5; 8.9) | 1.3  (−2.9; 5.5) |
| Adolescent relationship with caregiver [OM=6] |  |  |  | |  |  |  |  |  |  |
| Biological mother | Ref | Ref | Ref | | Ref | Ref | Ref | Ref | Ref | Ref |
| Biological father | 1.7  (−2.4; 5.9) | 0.3  (−4.9; 5.5) | **7.0***  (−3.2; 17.2) | | **10.5***  (−0.2; 21.3) | 0.02  (−4.5; 4.6) | −0.7  (−6.0; 4.5) | 2.5  (−3.3; 8.3) | 1.3  (−5.7; 8.3) | 0.03  (−4.8; 4.9) |
| Grandparent | **−13.0***** (−18.2;−7.8) | **−23.9***** (−30.4;−17.4) | **−17.8***** (−30.5;−5.2) | | **−17.6**** (−30.9;−4.2) | **−6.4**** (−12.1;−0.8) | −3.2 (−9.7; 3.4) | **−13.0***** (−20.2;−5.8) | −**18.0***** (−26.7;−9.2) | 0.1  (−5.9; 6.1) |
| Other relative | −1.1  (−5.7; 3.5) | −3.5  (−9.3; 2.3) | 1.9  (−13.2; 9.5) | | − 0.4  (−12.4; 11.5) | 2.6 (−2.5; 7.6) | − 0.7  (−6.5; 5.2) | −1.41 (−7.9; 5.0) | −3.1 (−10.9; 4.7) | 1.1  (−4.3; 6.5) |
| Sibling | 6.1 | 5.6 | 10.7 | | 6.0 | **8.2*** | 5.5 | 10.4 | 0.7 | 2.0 |
|  | (−2.5; 14.7) | (−5.1; 16.3) | (−10.3; 31.7) | | (−16.1; 28.1) | (−1.2; 17.6) | (−5.3; 16.4) | (−1.5; 22.3) | (−13.7; 15.1) | (−8.0; 12.0) |
| Caregiver religion |  |  |  | |  |  |  |  |  |  |
| Christianity | Ref | Ref | Ref | | Ref | Ref | Ref | Ref | Ref | Ref |
| Islam | −0.9  (−4.9; 3.0) | 2.7  (−2.3; 7.6) | | 0.2  (−9.2; 9.5) | −0.3  (−10.2; 9.5) | −2.2  (−6.5; 2.1) | **−4.6***  (−9.4; 0.2) | 0.3 (−5.1;−5.6) | **−5.0***  (−11.5; 1.5) | **−3.9***  (−8.3; 0.4) |
| Traditional | 1.8  (−2.3; 5.9) | 3.2  (−2.1; 8.4) | | 3.3  (−6.5; 13.1) | 4.5  (−5.9; 14.8) | −0.3  (−4.8; 4.3) | **−4.0***  (−9.1; 1.0) | 0.4  (−5.2; 6.0) | 1.5  (−5.3; 8.3) | **4.9****  (0.3; 9.5) |
| **Supplementary Table 1 continued** | | | | | | | | | | |
| **Independent variables** | **Overall HRQoL** | **Physical functioning** | **Role Limitations due to physical health** | | **Role Limitations due to emotional problems** | **Energy/ fatigue** | **Emotional wellbeing** | **Social functioning** | **Pain** | **General health** |
| Caregiver religion |  |  |  | |  |  |  |  |  |  |
| Christianity | Ref | Ref | Ref | | Ref | Ref | Ref | Ref | Ref | Ref |
| Islam | −0.9  (−4.9; 3.0) | 2.7  (−2.3; 7.6) | | 0.2  (−9.2; 9.5) | −0.3  (−10.2; 9.5) | −2.2  (−6.5; 2.1) | **−4.6***  (−9.4; 0.2) | 0.3 (−5.1;−5.6) | **−5.0***  (−11.5; 1.5) | **−3.9***  (−8.3; 0.4) |
| Traditional | 1.8  (−2.3; 5.9) | 3.2  (−2.1; 8.4) | | 3.3  (−6.5; 13.1) | 4.5  (−5.9; 14.8) | −0.3  (−4.8; 4.3) | **−4.0***  (−9.1; 1.0) | 0.4  (−5.2; 6.0) | 1.5  (−5.3; 8.3) | **4.9****  (0.3; 9.5) |
| Caregiver age (years) | −**0.4*****  (−0.5;−0.2) | −**0.5*****  (−0.6;−0.3) | | −**0.6*****  (−0.9;−0.3) | −**0.5*****  (−0.8;−0.2) | −**0.3*****  (−0.4;−0.1) | −**0.2*****  (−0.4;−0.1) | −**0.2*****  (−0.4;−0.1) | −**0.4*****  (−0.6;−0.2) | −**0.1***  (−0.3; 0.01) |
| Caregiver marital status |  |  |  | |  |  |  |  |  |  |
| Never married | Ref | Ref | Ref | | Ref | Ref | Ref | Ref | Ref | Ref |
| Married | **−4.6***  (−10.9; 1.6) | 1.2  (−6.8; 9.2) | **−13.6*** (−28.6; 1.5) | | −10.1 (−26.0; 5.7) | **−10.2*****  (−17.1; −3.4) | −4.6 (−12.4; 3.3 ) | **−6.7***  (−15.3; 1.9) | −1.8 (−12.3; 8.7) | −2.8  (−9.9; 4.3) |
| Widowed/Divorced | **−10.2***** (−16.7;−3.7) | −4.9  (−13.3; 3.4) | **−21.5*****  (−37.2; −5.9) | | −**22.0*****  (−38.5; −5.6) | **−14.4*****  (−21.6; −7.3) | **−7.7***  (−15.8; 0.4) | **−10.4****  (−19.4;−1.5) | **−7.9***  (−18.8; 3.0) | **−6.4***  (−13.8; 1.0) |
| Caregiver educational level [OM=2] |  |  |  | |  |  |  |  |  |  |
| No formal education | Ref | Ref | Ref | | Ref | Ref | Ref | Ref | Ref | Ref |
| Primary | **2.9****  (0.1; 5.8) | **2.9***  (−0.8; 6.6) | **6.4***  (−0.5; 13.7) | | **4.9***  (−2.3; 12.2) | **4.2*****  (1.1; 7.3) | **3.5*** (−0.03; 6.9) | 2.3 (−1.7; 6.2) | **4.0***  (−0.8; 8.7) | **−2.5***  (−5.7; 0.8) |
| Secondary | **4.6****  (0.1; 9.3) | −1.8  (−7.8; 4.3) | **8.7***  (−2.5; 20.0) | | 2.1 (−9.8; 14.0) | **10.0*****  (4.9; 15.0) | **12.3*****  (6.6; 18.0) | **9.9*****  (3.5; 16.3) | **7.5***  (−0.3; 15.3) | −0.5 (−5.9; 4.8) |
| Tertiary | **13.0*****  (5.7; 20.2) | 4.1 (−5.3; 13.4) | **25.2*****  (7.7; 42.6) | | **26.7*****  (8.3; 45.1) | **18.1*****  (10.3; 26.0) | **17.1*****  (8.3; 26.0) | **9.3***  (−0.6; 19.3) | **12.9****  (0.8; 25.0) | **7.6***  (− 06;15.9) |
| Caregiver BMI [OM=3] | **0.3****  (0.01; 0.5) | −0.1  (−0.5; 0.2) | **0.8****  (0.2; 1.4) | | **0.7****  (0.1; 1.4) | **0.4****  (0.1; 0.6) | **0.4*****  (0.1; 0.7) | 0.2  (−0.2; 0.5) | −0.2  (−0.6; 0.2) | **0.4*****  (0.1; 0.7) |
| Caregiver depressive symptoms (PHQ-9 scores) | −**2.2*****  (−2.5;−1.9) | −**1.4*****  (−1.9; −1.0) | −**4.1*****  (−4.8; −3.3) | | **−4.2*****  (−5.1; −3.4) | −**1.7*****  (−2.1; −1.4) | **−2.5*****  (−2.9; −2.1) | −**1.9*****  (−2.3; −1.4) | −**2.5*****  (−3.1; −2.0) | **−1.3*****  (−1.7; −0.9) |
| **Supplementary Table 1 continued** | | | | | | | | | | |
| **Independent variables** | **Overall HRQoL** | **Physical functioning** | **Role Limitations due to physical health** | | **Role Limitations due to emotional problems** | **Energy/ fatigue** | **Emotional wellbeing** | **Social functioning** | **Pain** | **General health** |
| Caregiver parenting stress (pss scores) | −**0.3***** (−0.5;−0.2) | −**0.2**** (−0.4;−0.1) | −**0.7***** (−1.0;−0.3) | | **−0.5***** (−0.9;−0.2) | −**0.5***** (−0.7;−0.4) | **−0.5***** (−0.6;−0.3) | −**0.4***** (−0.5;−0.2) | −**0.4***** (−0.6;−0.2) | 0.1  (−0.1; 0.2) |
| Caregiver socioeconomic status score | **1.8*****  (1.0; 2.7) | **0.7***  (−.0.4; 1.8) | **3.0*****  (1.0; 5.0) | | **4.2*****  (2.1; 6.3) | **2.5***** (1.7; 3.4) | **2.5***** (1.5; 3.5) | **1.3**** (0.14; 2.4) | **1.5**** (0.1; 2.9) | **1.0****  (0.1; 2.0) |
| Caregiver occupation [OM=24] |  |  |  | |  |  |  |  |  |  |
| Farmer | Ref | Ref | Ref | | Ref | Ref | Ref | Ref | Ref | Ref |
| Small scale Trader | **2.7***  (−0.2;5.5) | 2.3  (−1.3; 6.0) | **6.8***  (−0.1; 13.6) | | 4.6  (−2.6; 11.8) | **3.5****  (0.4; 6.6) | 0.9  (−2.6; 4.4) | **4.1****  (0.2;8.0) | **4.4***  (−0.4; 9.1) | **−**1.5  (−4.7; 1.7) |
| Casual labourer | 2.7  (−1.4; 6.9) | 2.9  (−2.5; 8.2) | **9.8***  (−0.1; 19.8) | | 1.1  (−9.4; 11.6) | 0.2  (−4.4; 4.7) | **5.3****  (0.1; 10.4) | **6.4****  (0.7; 12.0) | **6.2***  (−0.7; 13.1) | −**6.3****  (−10.9; −1.6) |
| Professional | **11.1*****  (4.0; 18.1) | **9.0****  (0.1; 18.0) | **18.2****  (1.3; 35.1) | | **20.5****  (2.6; 38.3) | **14.8***** (7.1; 22.5) | **11.1**** (2.4; 19.8) | 4.2  (−5.5; 13.8) | **10.7***  (−1.0; 22.4) | 4.9  (−3.1; 12.8) |
| **Notes**: ***** p<0.20; ****** p<0.05; ******* p<0.01; **Bolded** – some level of significance; **OM** – Missing observations; **HRQoL** – Health-related Quality of Life; **BMI** – Body Mass Index; **MUAC** – Mid upper arm circumference | | | | | | | | | | |
