# Supplementary material for: Correlates of health-related quality of life in primary caregivers of perinatally HIV infected and HIV exposed uninfected adolescents at the Kenyan Coast
Source: Health Qual Life Outcomes. 2022 Jan 21;20:11. doi: 10.1186/s12955-022-01915-z (PMC8780308; doi:10.1186/s12955-022-01915-z)
Supplement: Supplementary file 2 — Additional file 2: Table S2. Univariable linear regression analysis of the correlates of HRQoL among primary caregivers of adolescents living with HIV. Table S3. Univariable linear regression analysis of correlates of HRQoL among primary caregivers of adolescents perinatally exposed to HIV but uninfected. Table S4. Univariable linear regression analysis of the correlates of HRQoL among primary caregivers of HIV unexposed and uninfected adolescents. [file 12955_2022_1915_MOESM2_ESM.docx]

**Supplementary Table 2. Univariable linear regression analysis of the correlates of HRQoL among primary caregivers of adolescents living with HIV**

|  | **β-coefficient (95% CI) of HRQoL domains and overall scale as dependent variables** | | | | | | | | | |
| --- | --- | --- | --- | --- | --- | --- | --- | --- | --- | --- |
| Independent variables | Overall HRQoL | Physical functioning | Role Limitations due to physical health | | Role Limitations due to emotional problems | Energy/ fatigue | Emotional wellbeing | Social functioning | Pain | General health |
| Participating adolescent’s age [OM=1] | 0.05 (−1.4;1.5) | −1.2  (−3.2; 0.8) | 0.8  (−2.7; 4.3) | | 0.2  (−3.4; 3.8) | 0.7  (-0.8;2.3) | **0.8***  (−0.9; 2.5) | 0.9  (−1.0; 2.8) | −1.2  (−3.5; 1.1) | 0.8  (−0.9; 2.4) |
| Participating adolescent's sex [OM=1] |  |  |  | |  |  |  |  |  |  |
| Female | Ref | Ref | Ref | | Ref | Ref | Ref | Ref | Ref | Ref |
| Male | −1.8 (−6.2;2.6) | −1.7  (−7.7; 4.3) | −4.9  (−15.4; 5.6) | | −2.6  (−13.4; 8.1) | **−3.7***  (−8.3; 1.0) | 0.4  (−4.6; 5.4) | 0.8  (−4.9; 6.5) | -0.4  (−7.4; 6.6) | −1.1 (−6.2; 3.9) |
| Participating adolescent’s MUAC [OM=10] | 0.4  (−0.4; 1.2) | -0.3  (−1.3; 0.8) | **1.5***  (−0.3; 3.3) | | **1.2***  (−0.6; 3.0) | **0.9****  (0.1; 1.7) | −0.1  (−1.0; 0.8) | 0.1  (−0.9; 1.1) | −0.1  (−1.3; 1.1) | **0.7***  (−0.1; 1.6) |
| Participating adolescent's head circumference  [OM=10] | **1.7****  (0.3; 3.1) | 0.4  (−1.5; 2.3) | **2.5***  (−0.8; 5.8) | | **4.2****  (0.8; 7.5) | **2.2*****  (0.7; 3.7) | **1.1***  (−0.5; 2.7) | **2.9*****  (1.2; 4.7) | 2.1*****  (−0.2; 4.3) | **2.0****  (0.4; 3.6) |
| Participating adolescent’s number of years in school [OM=8] | **0.7***  (−0.3; 1.7) | −0.7  (−2.1; 0.8) | **2.7****  (0.3; 5.1) | | **1.9***  (−0.6; 4.4) | **1.4*****  (0.4; 2.5) | **0.9***  (−0.3; 2.0) | **1.3****  (0.03; 2.6) | 1.4*****  (−0.2; 3.0) | 0.2  (−1.0; 1.3) |
| Grade retention by Participating adolescent [OM=3] |  |  |  | |  |  |  |  |  |  |
| No | Ref | Ref | Ref | | Ref | Ref | Ref | Ref | Ref | Ref |
| Yes | 0.1  (−4.3; 4.6) | −0.7  (−6.8; 5.4) | −0.5  (−11.1; 10.2) | | −1.8  (−12.6; 9.0) | 0.1  (−4.7; 4.8) | 1.5  (−3.6; 6.5) | 1.5  (−4.1; 7.1) | −4.0  (−11.0; 3.0) | 2.5  (−2.5; 7.6) |
| Participating adolescent's orphan status |  |  |  | |  |  |  |  |  |  |
| Both parents alive | Ref | Ref | Ref | | Ref | Ref | Ref | Ref | Ref | Ref |
| One parent alive | **−3.3*** (−8.2;1.7) | **−2.8**  (−9.5;3.9) | **−9.4*** (−21.1;2.3) | | −5.4 (−17.5;6.6) | −0.7  (−6.0;4.6) | −1.7 (−7.4; 4.0) | **−4.5*** (−10.8; −1.8) | **−9.2**** (−16.9; -1.5) | 1.7  (−3.9; 7.2) |
| Both parents deceased | **−3.7*** (−9.8;2.3) | **−9.3**** (−17.5;−1.2) | **−10.5*** (−24.7; −3.7) | | −8.6  (−23.2; 6.0) | 3.7 (−2.7; 10.1) | 2.4 (−4.5; 9.2) | **−9.5**** (−17.1;−1.9) | **−9.6**** (−19.0;−0.3) | 8.4******  (1.7; 15.2) |
| Caregiver HIV Status |  |  |  | |  |  |  |  |  |  |
| Seronegative | Ref | Ref | Ref | | Ref | Ref | Ref | Ref | Ref | Ref |
| Seropositive | 0.8  (−3.8; 5.5) | **5.0*** (−1.3; 11.3) | **1.6**  (−8.3; 13.6) | | 0.1 (−11.2; 11.3) | −2.8 (−7.8; 2.1) | −0.2 (−5.5; 5.1) | 1.7 (−4.2; 7.7) | 0.2  (−7.1; 7.5) | **−4.2***  (−9.5; 1.0) |
|  |  |  |  | |  |  |  |  |  |  |
| **Supplementary Table 2 continued** | | | | | | | | | | |
| **Independent variables** | **Overall HRQoL** | **Physical functioning** | **Role Limitations due to physical health** | | **Role Limitations due to emotional problems** | **Energy/ fatigue** | **Emotional wellbeing** | **Social functioning** | **Pain** | **General health** |
| Caregiver sex |  |  |  | |  |  |  |  |  |  |
| Female | Ref | Ref | Ref | | Ref | Ref | Ref | Ref | Ref | Ref |
| Male | 1.1  (−5.3; 7.5) | −0.2  (−8.9; 8.5) | 1.7  (−13.4; 16.8) | | 6.8  (-8.6; 22.3) | 3.6  (−3.2; 10.4) | -0.4  (−7.7; 6.8) | 4.2  (−4.0; 12.3) | 0.6  (−9.4; 10.7) | -1.3  (−8.6; 5.9) |
| Adolescent relationship with caregiver [OM=5] |  |  |  | |  |  |  |  |  |  |
| Biological mother | Ref | Ref | Ref | | Ref | Ref | Ref | Ref | Ref | Ref |
| Biological father | -0.3  (−7.6; 7.0) | -1.2  (−11.0; 8.7) | 2.2  (−15.5; 19.9) | | 5.4  (−12.8; 23.7) | 3.4  (−4.1; 10.8) | −4.7  (−13.2; 3.7) | 1.9  (−7.6; 11.3) | 0.3  (−11.4; 12.1) | -2.0  (−10.5; 6.5) |
| Grandparent | **−10.6***** (−17.6;−3.6) | **−20.9***** (−30.4;−11.5) | **−17.0**** (−34.0;0.04) | | −10.3 (−27.9;7.2) | **−4.9***  (−12.1;2.3) | −2.4 (−10.5; 5.8) | **−15.7***** (−24.8;−6.7) | −**15.3***** (−26.5;−4.0) | 4.3  (−3.9; 12.4) |
| Other relative | −1.3  (−7.4; 4.8) | −4.4  (−12.6; 3.8) | -2.2  (−17.0; 12.7) | | − 1.2  (−16.5; 14.1) | 3.1 (−3.2; 9.3) | − 1.4  (−8.5; 5.7) | −4.4 (−12.3; 3.5) | −2.6 (−12.5; 7.2) | 3.5  (−3.6; 10.6) |
| Sibling | 5.5 | 6.6 | 11.8 | | 6.2 | 6.0 | 0.5 | **9.5*** | 1.6 | 4.3 |
|  | (−4.9; 15.9) | (−7.5; 20.6) | (−13.4; 37.0) | | (−19.9; 32.2) | (−4.7; 16.6) | (−12.6; 11.6) | (−3.9; 22.9) | (−15.2; 18.3) | (−7.8; 16.4) |
| Caregiver religion |  |  |  | |  |  |  |  |  |  |
| Christianity | Ref | Ref | Ref | | Ref | Ref | Ref | Ref | Ref | Ref |
| Islam | −1.2  (−7.9; 5.5) | 1.4  (−7.6; 10.5) | | -8.1  (−23.8; 7.6) | 0.9  (−15.2; 17.1) | 2.9  (−4.2; 10.0) | −2.6  (−10.2; 5.0) | 3.1 (−5.4;−11.7) | −5.6  (−16.0; 4.8) | −3.8  (−11.3; 3.7) |
| Traditional | 0.3  (−8.1; 8.7) | 2.3  (−9.0; 13.7) | | -3.8  (−23.5; 15.9) | -3.9  (−24.2; 16.3) | −22.2  (−11.1; 6.7) | −2.1  (−11.6; 7.4) | 2.3  (−8.4; 13.0) | 4.0  (−17.1; 9.1) | **7.4***  (-2.0; 16.8) |
| Caregiver age (years) [OM=2] | −**0.4*****  (−0.6;−0.3) | −**0.6*****  (−0.8;−0.3) | | −**0.8*****  (−1.2;−0.4) | −**0.7*****  (−1.1;−0.2) | −**0.4*****  (−0.6;−0.2) | −**0.2***  (−0.4; 0.03) | −**0.3*****  (−0.5;−0.1) | −**0.5*****  (−0.8;−0.2) | −**0.2***  (−0.4; 0.04) |
| Caregiver marital status |  |  |  | |  |  |  |  |  |  |
| Never married | Ref | Ref | Ref | | Ref | Ref | Ref | Ref | Ref | Ref |
| Married | **−9.6****  (−17.5; -1.7) | -4.5  (−15.4; 6.4) | **−22.2**** (−40.9; -3.5) | | −**22.9****  (−42.1; -3.8) | **−12.8*****  (−21.0; −4.5) | −4.5 (−13.6; 4.7 ) | **−10.4****  (−20.6; -0.3) | −4.7 (−17.2; 7.8) | −**9.0***  (−18.1; 0.3) |
| Widowed/Divorced | **−11.2***** (−19.4;−2.9) | −5.8  (−17.2; 5.6) | **−22.6****  (−42.2; −3.0) | | −**26.1*****  (−46.1; −6.1) | **−15.9*****  (−24.5; −7.2) | −4.8  (−14.4; 4.7) | **−14.2*****  (−24.8;−3.6) | **−11.8***  (−24.9; 1.2) | **−8.0***  (−17.5; 1.4) |
|  |  |  | |  |  |  |  |  |  |  |
| **Supplementary Table 2 continued** | | | | | | | | | | |
| **Independent variables** | **Overall HRQoL** | **Physical functioning** | **Role Limitations due to physical health** | | **Role Limitations due to emotional problems** | **Energy/ fatigue** | **Emotional wellbeing** | **Social functioning** | **Pain** | **General health** |
| Caregiver educational level |  |  |  | |  |  |  |  |  |  |
| No formal education | Ref | Ref | Ref | | Ref | Ref | Ref | Ref | Ref | Ref |
| Primary | **3.5***  (-1.6; 8.6) | **7.2****  (0.1; 14.4) | **11.2***  (−0.8; 23.3) | | 4.9 (−7.6; 17.4) | 2.1  (-3.2; 7.4) | -2.1 (−7.9; 3.7) | 3.4 (−3.2; 10.1) | **10.5****  (2.4; 18.6) | **−6.9****  (−12.7; 1.0) |
| Secondary | **10.5*****  (3.6; 17.5) | **7.3***  (−2.5; 17.1) | **26.5*****  (10.0; 43.0) | | **15.4***  (−1.7; 32.5) | **11.1*****  (3.9; 18.4) | **8.8****  (0.8; 16.7) | **12.4*****  (3.3; 21.6) | **17.6*****  (6.5; 28.6) | −1.0 (−9.0; 7.0) |
| Tertiary | **16.3*****  (7.6; 25.0) | **9.5*** (−2.7; 21.7) | **36.7*****  (16.1; 42.657.2) | | **34.8*****  (13.4; 56.1) | **20.5*****  (11.5; 29.6) | **12.4*****  (2.4; 22.3) | **7.5***  (−3.9; 19.0) | **20.0*****  (6.2; 33.8) | **7.9***  (− 2.1;17.9) |
| Caregiver BMI [OM=1] | **0.3***  (-0.05; 0.7) | −0.3  (−0.8; 0.2) | **0.8****  (-0.0; 1.7) | | **1.0****  (0.1; 1.8) | **0.5*****  (0.1; 0.9) | **0.5*****  (0.1; 0.9) | 0.03  (−0.4; 0.5) | −0.05  (−0.6; 0.5) | **0.6*****  (0.2; 1.0) |
| Caregiver depressive symptoms (PHQ-9 scores) | −**2.3*****  (−2.8;−1.7) | −**1.8*****  (−2.6; −1.0) | −**4.2*****  (−5.5; −2.9) | | **−3.4*****  (−4.8; −2.1) | −**2.0*****  (−2.5; −1.4) | **−2.2*****  (−2.8; −1.6) | −**2.2*****  (−2.9; −1.5) | −**2.4*****  (−3.2; −1.5) | **−1.4*****  (−2.0; −0.7) |
| Caregiver parenting stress (pss scores) | −**0.6***** (−0.8;−0.3) | −**0.4**** (−0.8;−0.1) | −**1.3***** (−1.8;−0.7) | | **−1.0***** (−1.5;−0.4) | −**0.6***** (−0.9;−0.4) | **−0.5***** (−0.7;−0.3) | −**0.5***** (−0.7;−0.2) | −**0.8***** (−1.1;−0.5) | -0.1  (−0.4; 0.2) |
| Caregiver socioeconomic status score | **2.9*****  (1.8; 4.0) | **1.2***  (−.0.4; 2.8) | **5.3*****  (2.5; 8.0) | | **6.5*****  (3.8; 9.3) | **3.5***** (2.3; 4.7) | **3.0***** (1.7; 4.3) | **1.6**** (0.1; 3.1) | **3.2**** (1.4; 5.0) | **2.4*****  (1.0; 3.7) |
| Caregiver occupation [OM=24] |  |  |  | |  |  |  |  |  |  |
| Farmer | Ref | Ref | Ref | | Ref | Ref | Ref | Ref | Ref | Ref |
| Small scale Trader | **6.9*****  (2.1;11.8) | **8.6****  (1.9; 15.3) | **16.5*****  (4.9; 28.0) | | **10.6***  (−1.5; 22.6) | **4.4***  (-0.7; 9.6) | -0.5  (−6.2; 5.2) | **7.5****  (1.2;13.8) | **11.2*****  (3.5; 18.8) | 1.5  (−4.2; 7.2) |
| Casual labourer | **8.0*****  (1.3; 14.6) | 7.5  (−1.6; 16.7) | **23.1*****  (7.4; 38.8) | | **12.8 ***  (−3.5; 29.2) | 2.0  (−4.9; 9.0) | 2.8  (-5.0; 10.6) | **13.9*****  (5.7; 22.5) | **18.3*****  (7.9; 28.8) | −1.8  (−9.5; 5.9) |
| Professional | **16.3*****  (7.6; 25.1) | **15.3****  (3.2; 27.4) | **29.5*****  (8.8; 50.3) | | **28.5****  (6.9; 50.1) | **21.0***** (11.8; 30.2) | **7.0***  (-3.2; 17.3) | **9.0***  (−2.4; 20.3) | **17.5*****  (3.8; 31.3) | 11.1******  (0.9; 21.3) |
| **Notes**: ***** p<0.20; ****** p<0.05; ******* p<0.01; **Bolded** – some level of significance; **OM** – Missing observations; **HRQoL** – Health-related Quality of Life; **BMI** – Body Mass Index; **MUAC** – Mid upper arm circumference | | | | | | | | | | |

**Supplementary Table 3. Univariable linear regression analysis of correlates of HRQoL among primary caregivers of adolescents perinatally exposed to HIV but uninfected**

|  | **β-coefficient (95% CI) of HRQoL domains and overall scale as dependent variables** | | | | | | | | | |
| --- | --- | --- | --- | --- | --- | --- | --- | --- | --- | --- |
| Independent variables | Overall HRQoL | Physical functioning | Role Limitations due to physical health | | Role Limitations due to emotional problems | Energy/ fatigue | Emotional wellbeing | Social functioning | Pain | General health |
| Participating adolescent’s age [OM=1] | **1.6***  (−0.4;3.6) | 1.2  (−1.7; 4.1) | 2.0  (−2.5; 6.6) | | 3.1  (−2.0; 8.2) | **3.0*****  (0.8;5.1) | 1.2  (−1.1; 3.5) | 0.9  (−1.6; 3.5) | −0.1  (−3.1; 3.0) | 1.4  (−0.9; 3.6) |
| Participating adolescent's sex [OM=1] |  |  |  | |  |  |  |  |  |  |
| Female | Ref | Ref | Ref | | Ref | Ref | Ref | Ref | Ref | Ref |
| Male | −3.2  (−8.8; 2.5) | **−5.6***  (−13.7; 2.4) | −3.0  (−15.7; 9.8) | | −5.7  (−14.0; 8.4) | **−**1.6  (−7.7; 4.5) | -2.5  (−8.9; 3.8) | 0.7  (−6.4; 7.7) | 0.3  (−8.3; 8.9) | −2.0  (−8.2; 4.3) |
| Participating adolescent’s MUAC [OM=5] | 0.7  (−0.5; 1.8) | **-1.3***  (−0.3; 2.9) | 1.1  (−1.5; 3.6) | | 1.0  (−1.9; 3.8) | 0.7  (-0.5; 2.0) | 0.3  (−1.0; 1.6) | -0.2  (−1.6; 1.2) | −0.3  (−2.0; 1.4) | -0.1  (−1.3; 1.2) |
| Participating adolescent's head circumference  [OM=3] | **1.3***  (-0.5; 3.1) | 1.3  (−1.2; 3.9) | 1.8  (−2.2; 5.8) | | **2.9***  (-1.5; 7.4) | **2.1****  (0.2; 4.0) | **1.5***  (−0.5; 3.5) | 0.01  (-2.2; 2.2) | 1.4  (−1.3; 4.1) | -0.3  (-2.3; 1.7) |
| Participating adolescent’s number of years in school [OM=3] | 0.4  (−0.9; 1.8) | 0.5  (−1.5; 2.4) | 0.1  (-2.9; 3.1) | | 1.2  (−2.2; 4.6) | 0.9  (-0.6; 2.4) | 0.4  (−1.1; 1.9) | 0.5  (-1.2; 2.2) | -0.2  (−2.3; 1.8) | -0.1  (−1.6; 1.4) |
| Grade retention by Participating adolescent [OM=2] |  |  |  | |  |  |  |  |  |  |
| No | Ref | Ref | Ref | | Ref | Ref | Ref | Ref | Ref | Ref |
| Yes | -2.0  (−7.9; 3.8) | −0.4  (−8.9; 8.0) | −3.9  (−17.0; 9.2) | | −4.1  (−18.7; 10.6) | -2.6  (−9.0; 3.7) | -2.8  (−9.4; 3.9) | 5.4*****  (−1.8; 12.7) | −4.7  (−13.6; 4.3) | -3.4  (−10.0; 3.1) |
| Participating adolescent's orphan status |  |  |  | |  |  |  |  |  |  |
| Both parents alive | Ref | Ref | Ref | | Ref | Ref | Ref | Ref | Ref | Ref |
| One parent alive | **−4.0*** (−9.7;1.7) | −2.8  (−10.6;5.0) | **−**3.9  (−17.1;9.2) | | −4.2  (−18.9;10.4) | **−5.4***  (−11.7;0.9) | −**5.4*** (−11.9; 1.2) | **−5.0*** (−12.3; 2.3) | 2.7  (−11.6; 6.1) | -2.8  (−9.3; 3.7) |
| Both parents deceased | **−23.8***** (−41.9;-5.6) | **−53.6***** (−78.3;−29.9) | **−**19.5  (−61.2; 22.2) | | −15.3  (−61.8; 31.2) | -8.2 (−28.2; 11.9) | **-13.9*** (−34.6; 6.8) | **−**13.9 (−36.9; 9.3) | **−24.7*** (−52.8; 3.4) | -5.1  (-25.7; 15.5) |
| **Supplementary Table 3 continued** | | | | | | | | | | |
| **Independent variables** | **Overall HRQoL** | **Physical functioning** | **Role Limitations due to physical health** | | **Role Limitations due to emotional problems** | **Energy/ fatigue** | **Emotional wellbeing** | **Social functioning** | **Pain** | **General health** |
| Participating adolescent's orphan status |  |  |  | |  |  |  |  |  |  |
| Both parents alive | Ref | Ref | Ref | | Ref | Ref | Ref | Ref | Ref | Ref |
| One parent alive | **−4.0*** (−9.7;1.7) | −2.8  (−10.6;5.0) | **−**3.9  (−17.1;9.2) | | −4.2  (−18.9;10.4) | **−5.4***  (−11.7;0.9) | −**5.4*** (−11.9; 1.2) | **−5.0*** (−12.3; 2.3) | 2.7  (−11.6; 6.1) | -2.8  (−9.3; 3.7) |
| Both parents deceased | **−23.8***** (−41.9;-5.6) | **−53.6***** (−78.3;−29.9) | **−**19.5  (−61.2; 22.2) | | −15.3  (−61.8; 31.2) | -8.2 (−28.2; 11.9) | **-13.9*** (−34.6; 6.8) | **−**13.9 (−36.9; 9.3) | **−24.7*** (−52.8; 3.4) | -5.1  (-25.7; 15.5) |
| Caregiver HIV Status |  |  |  | |  |  |  |  |  |  |
| Seronegative | Ref | Ref | Ref | | Ref | Ref | Ref | Ref | Ref | Ref |
| Seropositive | -1.9  (−8.5; 4.7) | 2.2 (−7.1; 11.5) | **-12.9***  (−27.5; 1.7) | | -8.5 (−24.9; 7.9) | 1.8 (−5.6; 8.7) | 1.3 (−6.2; 8.7) | -3.7 (−11.9; 4.5) | -3.6  (−13.7; 6.4) | **−**3.3  (−10.6; 4.0) |
| Caregiver sex |  |  |  | |  |  |  |  |  |  |
| Female | Ref | Ref | Ref | | Ref | Ref | Ref | Ref | Ref | Ref |
| Male | 3.3  (−5.9; 12.6) | 1.2  (−11.9; 14.3) | 9.6  (−11.1; 30.3) | | 14.5  (-8.4; 37.5) | **-0.2***  (−0.5; 0.1) | -2.7  (−13.1; 7.7) | **9.0***  (−2.4; 20.5) | 15.8******  (1.9; 29.6) | 1.6  (−8.7; 11.8) |
| Adolescent relationship with caregiver [OM=5] |  |  |  | |  |  |  |  |  |  |
| Biological mother | Ref | Ref | Ref | | Ref | Ref | Ref | Ref | Ref | Ref |
| Biological father | 5.4  (−4.8; 15.6) | 7.3  (−6.9; 21.5) | **18.2***  (−5.0; 41.5) | | **26.3****  (0.9; 51.8) | **-7.6***  (−18.9; 3.7) | −5.9  (−17.7; 5.8) | 4.1  (−8.8; 17.0) | **16.2****  (0.8; 31.6) | -0.3  (−12.0; 11.3) |
| Grandparent | **−12.2**** (−22.4;−2.0) | **−24.7***** (−38.9;−10.5) | -9.3 (−32.5;14.0) | | **−17.0*** (−42.5;8.5) | **−**6.6  (−17.9; 4.7) | −5.9 (−17.7; 5.8) | **−9.6*** (−22.5; 3.3) | −**16.6**** (−32.0;−1.2) | 2.7  (−9.0; 14.3) |
| Other relative | **12.4***  (−3.3; 28.1) | 9.3  (−12.5; 31.2) | **25.7***  (−10.0; 61.5) | | **33.0***  (−6.2; 72.2) | 7.4 (−10.0; 24.8) | 1.7  (−16.4; 19.8) | **18.5*** (−1.3; 38.3) | **15.3*** (−8.4; 39.0) | 10.4  (−7.5; 28.3) |
| Sibling | 14.7 | 9.3 | 25.7 | | 33.0 | 19.9 | **23.7*** | -15.9 | -17.8 | 16.7 |
|  | (−16.2;45.6) | (−33.8; 52.4) | (−44.8; 96.3) | | (−44.3; 110.3) | (−14.3; 54.1) | (−12.0; 59.4) | (−55.0; 23.2) | (−64.6; 28.9) | (−18.6; 51.9) |
| Caregiver religion |  |  |  | |  |  |  |  |  |  |
| Christianity | Ref | Ref | Ref | | Ref | Ref | Ref | Ref | Ref | Ref |
| Islam | 2.1  (−5.5; 9.8) | **8.9***  (−1.9; 19.6) | | **11.2***  (-5.8; 28.2) | -3.4  (−22.5; 15.6) | -5.0  (−13.3; 3.2) | −4.7  (−13.2; 3.8) | 4.5  (−4.9; 14.0) | −1.3  (−12.9; 10.3) | −2.0  (−10.4; 6.4) |
| Traditional | -1.3  (−11.9; 9.2) | 2.5  (−12.3; 17.2) | | -4.5  (−27.9; 19.0) | -6.8  (−33.1; 19.5) | −0.8  (−12.2; 10.6) | −6.5  (−18.3; 5.3) | -7.0  (−20.0; 6.1) | -1.2  (−17.2; 14.9) | 5.1  (-6.5; 16.7) |
| **Supplementary Table 3 continued** | | | | | | | | | | |
| **Independent variables** | **Overall HRQoL** | **Physical functioning** | **Role Limitations due to physical health** | | **Role Limitations due to emotional problems** | **Energy/ fatigue** | **Emotional wellbeing** | **Social functioning** | **Pain** | **General health** |
| Caregiver age (years) [OM=2] | −**0.3****  (−0.6;−0.05) | −**0.6*****  (−1.0;−0.2) | | -0.3  (−0.9;0.3) | −0.01  (−0.7; −0.7) | −**0.2***  (−0.5; 0.1) | −**0.4****  (−0.7; 0.1) | −**0.3***  (−0.7; 0.01) | −**0.3***  (−0.7; 0.1) | −0.01  (−0.3; 0.3) |
| Caregiver marital status |  |  |  | |  |  |  |  |  |  |
| Never married | Ref | Ref | Ref | | Ref | Ref | Ref | Ref | Ref | Ref |
| Married | 5.6  (−10.0;21.2) | 10.7  (−11.6; 33.3) | **−**3.9  (−39.4; 31.6) | | 10.0 (−30.0; 49.9) | 2.2 (−15.0; 19.4) | 1.5 (−16.4; 19.3 ) | **17.0***  (−2.8; 36.9) | 8.7 (−15.9; 33.3) | −2.0  (−10.4; 6.4) |
| Widowed/Divorced | −4.4 (−20.0;−11.3) | 0.7  (−22.0; 23.4) | **−**21.3 (−57.0; −14.4) | | −6.4 (−46.6; −33.7) | **−**6.8 (−24.0; 10.5) | −7.1  (−25.0; 10.9) | 8.7 (−11.2; 28.7) | 1.0  (−23.7; 25.7) | 5.1  (−6.5; 16.7) |
| Caregiver educational level |  |  |  | |  |  |  |  |  |  |
| No formal education | Ref | Ref | Ref | | Ref | Ref | Ref | Ref | Ref | Ref |
| Primary | **4.8***  (-1.2; 10.8) | 2.9  (-5.5; 11.4) | 6.7  (−6.8; 20.2) | | **13.1***  (−1.8; 28.1) | **6.5****  (-0.1; 13.1) | 5.7*****  (−1.1; 12.6) | 1.8 (−5.9; 9.5) | 1.6  (-7.7; 11.0) | 1.6  (−5.3; 8.4) |
| Secondary | **8.5***  (-19.4; 2.4) | **-18.9****  (−34.3; -3.5) | **23.4***  (-48.3; 0.8) | | **-19.2***  (−46.4; 8.0) | 5.0  (-7.0; 17.0) | **8.1***  (-4.4; 20.6) | 2.5  (-16.5; 11.5) | -7.2  (-24.2; 9.8) | −1.1 (−13.5; 11.3) |
| Caregiver BMI [OM=2] | 0.4  (-0.2; 1.0) | 0.3  (−05; 1.2) | **1.5****  (0.2; 2.8) | | 0.8  (-0.7; 2.3) | **0.4***  (-0.3; 1.0) | 0.1  (-0.6; 0.8) | 0.1  (−0.7; 0.8) | **−0.6***  (−1.6; 0.3) | 0.1  (-0.6; 0.8) |
| Caregiver depressive symptoms (PHQ-9 scores) | −**2.3*****  (−2.9;−1.7) | −**1.6*****  (−2.6; −0.6) | −**3.8*****  (−5.3; −2.3) | | **−5.1*****  (−6.7; −3.6) | −**1.9*****  (−2.7; −1.2) | **−2.2*****  (−2.9; −1.4) | −**1.8*****  (−2.1; −0.3) | −**2.6*****  (−3.6; −1.5) | **−1.6*****  (−2.3; −0.8) |
| Caregiver parenting stress (pss scores) | −**0.3***** (−0.6;−0.1) | −**0.3*** (−0.7;0.02) | −**0.4*** (−1.0;0.2) | | **−0.6*** (−1.2;−0.1) | −**0.6***** (−0.9;−0.3) | **−0.5***** (−0.8;−0.2) | −**0.3*** (−0.6;−0.04) | −0.1  (−0.5; 0.3) | 0.1  (−0.2; 0.4) |
| Caregiver socioeconomic status score | 0.5  (-1.7; 2.8) | 1.0  (−.2.2; 4.1) | 0.3  (-4.7; 5.3) | | 1.8  (-3.7; 7.4) | **1.9***  (-0.5; 4.3) | 1.0  (-1.5; 3.4) | -0.1 (-2.8; 2.7) | -0.4  (-3.8; 3.0) | **-1.8***  (-4.3; 0.6) |
| Caregiver occupation [OM=24] |  |  |  | |  |  |  |  |  |  |
| Farmer | Ref | Ref | Ref | | Ref | Ref | Ref | Ref | Ref | Ref |
| Small scale Trader | -1.1  (-7.3;5.2) | -1.1  (-9.9; 7.7) | -3.7  (-17.6; 10.2) | | -2.8  (−18.3; 12.7) | **4.8***  (-1.9; 11.4) | 3.0  (-4.0; 9.9) | 1.0  (-6.9;8.7) | 0.5  (-9.0; 9.9) | **-8.4****  (−15.1; -1.7) |
| Casual labourer | 1.5  (-6.9; 9.9) | 5.4  (−6.4; 17.3) | 6.7  (-12.0; 25.4) | | 4.4  (−25.4; 16.5) | -1.6  (−10.6; 7.4) | 7.4  (-2.0; 16.8) | 2.1  (-12.6; 8.3) | -2.3  (-15.1; 10.4) | **−8.5***  (−17.5; 0.5) |
| **Notes**: ***** p<0.20; ****** p<0.05; ******* p<0.01; **Bolded** – some level of significance; **OM** – Missing observations; **HRQoL** – Health-related Quality of Life; **BMI** – Body Mass Index; **MUAC** – Mid upper arm circumference | | | | | | | | | | |

**Supplementary Table 4. Univariable linear regression analysis of the correlates of HRQoL among primary caregivers of HIV unexposed and uninfected adolescents**

|  | **β-coefficient (95% CI) of HRQoL domains and overall scale as dependent variables** | | | | | | | | | |
| --- | --- | --- | --- | --- | --- | --- | --- | --- | --- | --- |
| Independent variables | Overall HRQoL | Physical functioning | Role Limitations due to physical health | | Role Limitations due to emotional problems | Energy/ fatigue | Emotional wellbeing | Social functioning | Pain | General health |
| Participating adolescent’s age [OM=1] | **-1.0***  (−2.1; 0.1) | **-0.8****  (−1.6; 0.02) | **-2.1***  (−5.2; 1.0) | | -1.5  (−4.6; 1.6) | 0.1  (-1.3;1.5) | -0.03  (−1.8; 1.8) | -0.7  (−2.6; 1.3) | **−2.5****  (−4.8; -0.2) | **-1.5****  (−2.7; -0.3) |
| Participating adolescent's sex [OM=1] |  |  |  | |  |  |  |  |  |  |
| Female | Ref | Ref | Ref | | Ref | Ref | Ref | Ref | Ref | Ref |
| Male | −0.3  (−3.9; 3.2) | -1.2  (−3.8; 1.3) | −1.9  (−11.7; 8.0) | | −1.2  (−11.1; 8.7) | **−**1.9  (−6.3; 2.6) | **4.2***  (−1.5; 9.9) | -1.5  (−7.8; 4.7) | -0.1  (−7.4; 7.3) | −0.2  (−4.0; 3.6) |
| Participating adolescent’s MUAC [OM=5] | -**0.8****  (−1.5; -0.1) | -0.3  (−0.8; 0.2) | **-1.4***  (−3.3; 0.5) | | **-2.3****  (−4.2; -0.4) | -0.2  (-1.1; 0.7) | **-0.9***  (−2.0; 0.2) | -0.5  (−1.7; 0.7) | **−1.2***  (−2.6; 0.2) | **-0.7***  (−1.4; 0.02) |
| Participating adolescent's head circumference  [OM=5] | -0.6  (-1.7; 0.4) | -0.5  (−1.3; 0.3) | -3.4******  (−6.3; -0.5) | | **-1.4**  (-4.4; 1.6) | 0.1  (-1.2; 1.5) | -0.3  (−2.0; 1.4) | 0.1  (-1.8; 2.0) | -0.8  (−3.0; 1.4) | -0.02  (-1.1; 1.1) |
| Participating adolescent’s number of years in school [OM=3] | -0.2  (−1.4; 1.0) | -0.04  (−0.9; 0.8) | -1.4  (-4.6; 1.8) | | -1.5  (−4.7; 1.8) | 0.4  (-1.1; 1.8) | 0.4  (−1.4; 2.3) | 1.2  (-0.8; 3.3) | -1.0  (−3.4; 1.4) | -0.4  (−1.6; 0.8) |
| Grade retention by Participating adolescent [OM=1] |  |  |  | |  |  |  |  |  |  |
| No | Ref | Ref | Ref | | Ref | Ref | Ref | Ref | Ref | Ref |
| Yes | 0.5  (−3.1; 4.1) | −0.5  (−3.1; 2.2) | 1.5  (−8.5; 11.4) | | 0.1  (−9.9; 10.1) | 2.5  (−2.1; 7.0) | 1.0  (−4.8; 6.7) | -2.8  (−9.1; 3.5) | 3.2  (−4.2; 10.5) | 0.8  (−3.0; 4.6) |
| Participating adolescent's orphan status [OM=1] |  |  |  | |  |  |  |  |  |  |
| Both parents alive | Ref | Ref | Ref | | Ref | Ref | Ref | Ref | Ref | Ref |
| One parent alive | 0.4  (−5.9; 6.7) | −0.9  (−5.5; 3.7) | **−**0.8  (−18.3;16.7) | | −4.2  (−21.8 ;13.4) | **−**0.6  (−8.7; 7.4) | 2.3 (−7.9; 12.4) | 0.9 (−10.3; 12.1) | **12.8***  (−0.1; 25.7) | -0.9  (−7.7; 5.9) |
| Caregiver HIV Status |  |  |  | |  |  |  |  |  |  |
| Seronegative | Ref | Ref | Ref | | Ref | Ref | Ref | Ref | Ref | Ref |
| Seropositive | 5.2  (−10.8; 21.2) | 2.4 (−9.3; 14.1) | 19.1  (−25.3; 63.4) | | 17.7 (−27.0; 62.4) | 3.8 (−16.6; 24.1) | -12.8 (−38.6; 12.9) | 3.7 (−24.7; 32.0) | 15  (−18.1; 48.1) | 9.6  (−7.5; 26.7) |
|  |  |  |  | |  |  |  |  |  |  |
| **Supplementary Table 4 continued** | | | | | | | | | | |
| **Independent variables** | **Overall HRQoL** | **Physical functioning** | **Role Limitations due to physical health** | | **Role Limitations due to emotional problems** | **Energy/ fatigue** | **Emotional wellbeing** | **Social functioning** | **Pain** | **General health** |
| Caregiver sex |  |  |  | |  |  |  |  |  |  |
| Female | Ref | Ref | Ref | | Ref | Ref | Ref | Ref | Ref | Ref |
| Male | 1.2  (−3.4; 5.7) | **-3.6****  (−6.9; -0.3) | 3.7  (−8.9; 16.3) | | 7.8  (-4.8; 20.5) | -0.1  (−5.9; 5.7) | **7.2***  (−0.1; 14.5) | 0.8  (−7.2; 8.9) | 2.9  (12.3; 6.5) | 1.4  (−3.5; 6.3) |
| Adolescent relationship with caregiver [OM=1] |  |  |  | |  |  |  |  |  |  |
| Biological mother | Ref | Ref | Ref | | Ref | Ref | Ref | Ref | Ref | Ref |
| Biological father | 1.3  (−3.8; 6.5) | **-2.6***  (−6.4; 1.2) | 6.3  (−8.2; 20.7) | | 6.6  (-8.0; 21.1) | 1.5  (−4.9; 7.9) | 4.2  (−4.2; 12.5) | 2.4  (−6.8; 11.5) | -4.9  (-15.6; 5.7) | 1.1  (−4.5; 6.7) |
| Other relative | -2.0  (−11.4; 7.4) | -3.9  (−10.8; 3.0) | -0.9  (−27.2; 25.3) | | 2.0  (−24.4; 28.4) | **8.0***  (-3.6; 19.6) | -6.0  (−21.2; 9.1) | -4.3 (−20.9; 12.4) | -7.4 (−26.8; 11.9) | -2.1  (−12.3; 8.1) |
| Sibling | **16.9*** | 1.9 | 19.9 | | 18.7 | **37.2***** | **28.0*** | **35.3*** | **31.7*** | 3.0 |
|  | (−5.6; 39.5) | (−14.6; 18.5) | (−43.1; 82.9) | | (−44.8; 82.2) | (9.3; 65.0) | (−8.3; 64.3) | (−4.7; 75.3) | (−14.8; 78.3) | (−27.4; 21.5) |
| Caregiver religion |  |  |  | |  |  |  |  |  |  |
| Christianity | Ref | Ref | Ref | | Ref | Ref | Ref | Ref | Ref | Ref |
| Islam | -3.5  (−9.2; 2.2) | -1.7  (−5.8; 2.5) | | -1.0  (-16.8; 14.9) | 2.4  (−13.6; 18.4) | **-6.4***  (−13.6; 0.8) | **−6.2***  (−15.4; 3.0) | **-8.9***  (−19.0; 1.2) | **−8.3***  (−20.1; 3.5) | **−5.4***  (−11.5; 0.7) |
| Traditional | 0.6  (−3.9; 5.1) | 0.04  (−3.3; 3.3) | | 5.8  (−6.7; 18.3) | 6.5  (−6.1; 19.2) | 0.1  (−5.6; 5.8) | **−5.4***  (−12.7; 1.8) | 1.3  (−6.6; 9.3) | 2.3  (−7.0; 11.6) | -0.4  (-5.3; 4.4) |
| Caregiver age (years) [OM=2] | −**0.1***  (−1.3; −0.1) | -0.04  (−0.2; 0.1) | | -0.3  (−0.8;0.2) | −0.3  (−0.8; 0.2) | −0.1  (−0.3; 0.1) | −**0.2***  (−0.5; 0.1) | −0.1  (−0.4; 0.2) | −0.2  (−0.6; 0.2) | 0.04  (−0.1; 0.2) |
| Caregiver marital status |  |  |  | |  |  |  |  |  |  |
| Never married | Ref | Ref | Ref | | Ref | Ref | Ref | Ref | Ref | Ref |
| Married | **-16.8***  (−39.3; 5.7) | -2.4  (−18.9; 14.2) | **−**19.0  (−81.8; 43.8) | | -16.9 (−80.1; 46.3) | -**36.5****  (−64.8; -8.3) | **28.0***  (−64.3; 8.3) | **-35.3***  (−75.1; 4.5) | **-33.2***  (−79.7; 13.3) | 3.0  (−21.3; 27.4) |
| Widowed/Divorced | **−16.2***  (−39.2; −6.8) | -2.9  (−19.8; 14.0) | **−**18.4 (−82.6; 45.8) | | −22.8 (−87.4; −41.8) | **−36.1****  (−64.9; -7.2) | **−24.0***  (−61.1; 13.1) | **33.6*** (−74.3; 7.2) | -27.4  (−75.0; 20.2) | 3.2  (−21.7; 28.0) |
| **Supplementary Table 4 continued** | | | | | | | | | | |
| **Independent variables** | **Overall HRQoL** | **Physical functioning** | **Role Limitations due to physical health** | | **Role Limitations due to emotional problems** | **Energy/ fatigue** | **Emotional wellbeing** | **Social functioning** | **Pain** | **General health** |
| Caregiver educational level |  |  |  | |  |  |  |  |  |  |
| No formal education | Ref | Ref | Ref | | Ref | Ref | Ref | Ref | Ref | Ref |
| Primary | **3.1***  (-0.5; 6.7) | 1.0  (-1.7; 3.7) | 4.7  (−5.5; 15.0) | | 3.8 (−6.5; 14.2) | **5.2****  (0.6; 9.7) | 7.8*******  (2.1; 13.4) | 1.3 (−5.0; 7.6) | 1.7  (-5.9; 9.3) | 1.2  (−2.4; 5.2) |
| Secondary | **10.3*****  (2.9; 17.7) | 3.2  (−2.4; 8.7) | **14.9***  (-6.2; 36.0) | | 6.8 (−14.6; 28.1) | **14.9*****  (5.5; 24.3) | **20.4*****  (8.7; 32.2) | **18.4*****  (5.3; 31.4) | **11.9***  (-3.8; 27.6) | 4.4  (−3.8; 12.6) |
| Tertiary | **14.2***  (-1.5; 29.9) | 3.2 (−8.7; 15.0) | 22.4  (-22.3; 67.1) | | 20.1 (25.2; 65.4) | **12.4***  (-7.4; 32.3) | **24.8***  (-0.1; 49.8) | **37.1*****  (9.5; 64.7) | **22.9***  (-10.3; 56.2) | 5.4  (− 11.9; 22.8) |
| Caregiver BMI [OM=2] | 0.2  (-0.3; 0.6) | -0.1  (−0.4; 0.2) | 0.3  (-0.9; 1.6) | | 0.3  (-0.9; 1.5) | 0.1  (-0.4; 0.7) | 0.2  (-0.5; 0.9) | **0.8****  (0.1; 1.6) | 0.2  (−0.7; 1.1) | 0.1  (-0.3; 0.6) |
| Caregiver depressive symptoms (PHQ-9 scores) | −**1.9*****  (−2.3; −1.4) | −**0.5*****  (−0.8; −0.1) | −**3.8*****  (−5.1; −2.6) | | **−4.0*****  (−5.2; −2.7) | −**1.2*****  (−1.8; −0.6) | **−3.2*****  (−3.8; −2.5) | −**2.2*****  (−3.0; −1.3) | −**2.5*****  (−3.5; −1.6) | **−0.7****  (−1.2; −0.1) |
| Caregiver parenting stress (pss scores) | −0.1 (−0.3;−0.1) | 0.02  (−0.1; 0.2) | −**0.1**  (−0.7 ; 0.5) | | −0.02 (−0.6;−0.6) | −**0.3***** (−0.6;−0.1) | **−0.5***** (−0.8;−0.2) | −**0.3*** (−0.7;−0.1) | **−0.4***  (−0.8; 0.1) | **0.2***  (−0.03; 0.4) |
| Caregiver socioeconomic status score | 1.2  (-0.1; 2.5) | 0.2  (−.0.7; 1.2) | 1.7  (-2.0; 5.3) | | **2.6***  (-1.0; 6.3) | **1.6***  (-0.04; 3.3) | **2.1***  (-0.03; 4.2) | **1.7*** (-0.6; 4.0) | 0.1  (-2.6; 2.9) | **0.9***  (-0.5; 2.3) |
| Caregiver occupation [OM=24] |  |  |  | |  |  |  |  |  |  |
| Farmer | Ref | Ref | Ref | | Ref | Ref | Ref | Ref | Ref | Ref |
| Small scale Trader | **3.6***  (-0.1; 7.4) | 1.3  (-1.4; 4.1) | 8.4*****  (-2.0; 18.8) | | **-9.4***  (−1.1; 19.8) | 2.1  (-2.7; 6.9) | 1.8  (-4.2; 7.8) | 3.1  (-3.6; 9.8) | 2.6  (-5.2; 10.4) | **3.5***  (−0.4; 7.5) |
| Casual labourer | 0.6  (-6.0; 7.3) | -0.9  (-5.8; 4.0) | -2.7  (-21.3; 15.8) | | -1.5  (−20.2; 17.1) | 1.9  (−6.7; 10.4) | **9.5***  (-1.2; 20.3) | 4.7  (-7.3; 16.7) | -2.7  (-11.3; 16.5) | **−6.5***  (−13.6; 0.6) |
| Professional | **7.5***  (-3.9; 18.9) | 2.9  (-5.5; 11.4) | 16.0  (-15.8; 47.8) | | **21.5***  (-10.4; 53.5) | 2.3  (-12.5; 17.0) | **17.2***  (-1.2; 35.6) | -1.1  (−21.6; 19.4) | 12.0  (12.0; 35.9) | 3.5  (-15.6; 8.6) |
| **Notes**: ***** p<0.20; ****** p<0.05; ******* p<0.01; **Bolded** – some level of significance; **OM** – Missing observations; **HRQoL** – Health-related Quality of Life; **BMI** – Body Mass Index; **MUAC** – Mid upper arm circumference | | | | | | | | | | |
