# Supplementary material for: Correlates of health-related quality of life in primary caregivers of perinatally HIV infected and HIV exposed uninfected adolescents at the Kenyan Coast
Source: Health Qual Life Outcomes. 2022 Jan 21;20:11. doi: 10.1186/s12955-022-01915-z (PMC8780308; doi:10.1186/s12955-022-01915-z)
Supplement: Supplementary file 3 — Additional file 3: Table S5. Description of the HRQoL measure (RAND SF-36). [file 12955_2022_1915_MOESM3_ESM.docx]

**Supplementary table 5**. Description of the HRQoL measure (RAND SF-36)

| **RAND SF-36 scale** | **Description** | **Number of Items** |
| --- | --- | --- |
| Physical functioning | Assess a person’s ability to engage in activities of daily life | 10 |
| Role limitations due to physical health | Assess the impact of an individual’s physical health on their ability to perform on the job, around the house etc | 4 |
| Role limitations due to emotional problems | Assess the impact of individual’s emotional health on their ability to perform around the house, on the job | 3 |
| Energy/fatigue | Assess a person’s energy levels and fatigue in performing various activities | 4 |
| Emotional well-being | Assess symptoms of mental health problems | 5 |
| Social functioning | Assess whether social activities have been limited health | 2 |
| Pain | Assess bodily pain and its interference with normal activities | 2 |
| General Health | Assess a person’s perception of their general health, resistance to illness and health outlook | 6 |
| **Scoring instructions of the tool**:  Scoring the tool follows a two-step process. Firstly, pre-coded numeric values are converted to percentages using a predetermined scoring key with a high score defining a more favorable state of health. In the second step, items in the same domain are averaged together to create the 8 different scale scores | | |
