# Supplementary material for: Correlates of health-related quality of life in primary caregivers of perinatally HIV infected and HIV exposed uninfected adolescents at the Kenyan Coast
Source: Health Qual Life Outcomes. 2022 Jan 21;20:11. doi: 10.1186/s12955-022-01915-z (PMC8780308; doi:10.1186/s12955-022-01915-z)
Supplement: Supplementary file 4 — Additional file 4. Graphical distribution of the mean HRQoL scores across the three groups of caregivers. [file 12955_2022_1915_MOESM4_ESM.docx]

Graphical distribution of the mean HRQoL scores across the three groups of careivers
